# Triaminoalkenes Derived from Cyclic (Alkyl)(amino)carbenes with a 1,1′‐Ferrocenylene Backbone and *N*‐Heterocyclic Carbenes: fcCAAC–NHC Heterodimers

**DOI:** 10.1002/open.202500156

**Published:** 2025-04-14

**Authors:** Suman Yadav, Clemens Bruhn, Clemens Lücke, Richard Rudolf, Tabea Pfister, Biprajit Sarkar, Ulrich Siemeling

**Affiliations:** ^1^ Institute of Chemistry University of Kassel Heinrich‐Plett‐Straße 40 34132 Kassel Germany; ^2^ Institut für Anorganische Chemie Universität Stuttgart Pfaffenwaldring 55 70569 Stuttgart Germany; ^3^ Institut für Chemie und Biochemie, Anorganische Chemie Freie Universität Berlin Fabeckstraße 34–36 14195 Berlin Germany

**Keywords:** alkenes, carbenes, iron, metallocenes, radicals

## Abstract

The study addresses triaminoalkenes derived from [3]ferrocenophane‐type cyclic (alkyl)(amino)carbenes (fcCAACs) fc(CPh_2_—C—NR) (fc = 1,1′‐ferrocenylene) and *N*‐heterocyclic carbenes (NHCs). Stable target compounds are obtained in good yields as crystalline solids by the combination of [fc(CPh_2_—CH=NMe)][BF_4_] with *N*,*N*′‐dimethylimidazolin‐2‐ylidene and of [fc(CPh_2_—CH=N‐*p*‐C_6_H_4_‐*t*Bu)](OTf) with 1,3,4,5‐tetramethylimidazolin‐2‐ylidene, respectively, followed by treatment of the resulting addition product with KN(SiMe_3_)_2_. Due to the presence of a triaminoethene unit and a ferrocene moiety, four redox states are expected for such fcCAAC–NHC heterodimers, viz., electroneutral, mono‐, di‐, and tricationic. An investigation of their redox behavior by electrochemical methods (cyclic voltammetry and differential pulse voltammetry) has revealed that these compounds undergo two consecutive one‐electron oxidations, with the poor stability of the dicationic species in solution preventing an observation of the tricationic redox state. A density functional theory (DFT) study shows that the highest occupied molecular orbital (HOMO) is localized on the *C*=CN_2_ atom, which, in agreement with electron paramagnetic resonance results, is the site of the first oxidation. The second oxidation mainly involves the Fe atom, where the HOMO−1 is localized, resulting in a species with a triplet ground state composed, to a first approximation, of a carbon‐centered and an iron‐centered radical.

## Graphical abstract

Nucleophilic addition of *N*‐heterocyclic carbenes (NHCs) to protonated ferrocene‐based cyclic (alkyl)(amino)carbenes (fcCAACs), followed by treatment with KN(SiMe_3_)_2_, affords fcCAAC–NHC heterodimers. Dicationic diradicals with a triplet ground state are formed by sequential oxidation of these ferrocenylene‐functionalized triaminoalkenes, but are too unstable in solution for observation of the tricationic redox state.

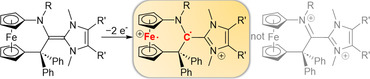

## 1 Introduction

**Figure 1:**
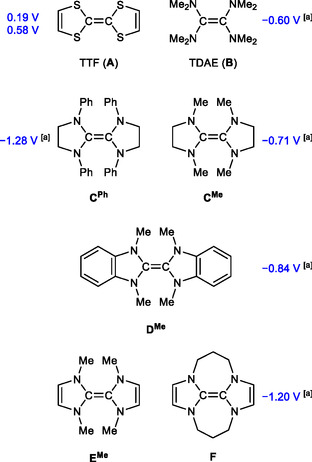
TTF (TTF, **A**), tetrakis(dimethylamino)ethene (TDAE, **B**) and closely related electron‐rich olefins, together with published electrochemical data (*E*_1/2_ vs SCE in DMF; see text for refs.). [a] Two‐electron process.

Electron‐rich olefins constitute a particularly important class of redox‐active compounds.^[^ ^1^, ^2^, ^3^, ^4^ ^]^ Iconic representatives are tetrathiafulvalene (TTF) and tetrakis(dimethylamino)ethene (TDAE) (**Figure** **1**). The synthesis and redox behavior of TTF (**A**) was first described in 1970,^[^ ^5^, ^6^, ^7^ ^]^ although a benzanellated congener had been known already since 1926.^[^ ^8^ ^]^ The discovery in the early 1970s that, due to its redox activity, TTF can form compounds behaving as organic solid‐state semiconductors^[^ ^9^ ^]^ or even exhibit metallic conductivity^[^ ^10^ ^]^ had a decisive impact on the then burgeoning field of organic metals,^[^ ^11^, ^12^, ^13^ ^]^ molecular electronics, and beyond.^[^ ^14^, ^15^, ^16^, ^17^, ^18^, ^19^, ^20^, ^21^, ^22^ ^]^ TDAE (**B**) was reported in 1950.^[^ ^23^ ^]^ Its redox behavior was studied in the early 1960s,^[^ ^24^, ^25^, ^26^ ^]^ paving the way to the application of TDAE as reducing agent in organic synthesis.^[^ ^1^, ^2^, ^3^, ^4^, ^27^, ^28^, ^29^, ^30^, ^31^, ^32^, ^33^ ^]^ The search for applications of **B** in the field of molecular materials resulted in the organic ferromagnet formed with C_60_.^[^ ^34^ ^]^ At the same time, cyclic analogues of TDAE became available, triggered by the quest for stable carbenes, which culminated in Arduengo's report of a stable crystalline *N*‐heterocyclic carbene (NHC) in 1991.^[^ ^35^, ^36^, ^37^ ^]^ The synthesis of the first example, **C** ^**Ph**^, was described by Wanzlick in 1961^[^ ^38^, ^39^, ^40^ ^]^ and its redox behavior was studied by Lemal already in 1962.^[^ ^41^ ^]^ Numerous tetraaminoethenes **C** ^**R**^ bearing *N*‐substituents R ≠ Ph were reported soon after.^[^ ^26^, ^42^, ^43^ ^]^ The conformational restriction due to the cyclic structural elements present in these compounds leads to a more efficient π‐interaction of the nitrogen lone pairs with the olefinic unit, which consequently is more electron‐rich in **C** ^**R**^ than in TDAE (**B**), as is reflected by the redox potentials of these compounds. The electrochemical oxidation of **B** in dimethylformamide (DMF) occurs as a reversible two‐electron step at *E*_1/2_ = −0.60 V versus saturated calomel electrode (SCE).^[^ ^44^, ^45^ ^]^ The most closely related cyclic analogue is **C** ^**Me**^, which exhibits a two‐electron oxidation at *E*_1/2_ = −0.71 V versus SCE in DMF,^[^ ^46^ ^]^ thus being a stronger reducing agent than **B**. The potential published for the two‐electron oxidation of the benzanellated congener **D** ^**Me**^ (*E*_1/2_ = −0.84 V vs SCE in DMF) shows that this compound is an even stronger reductant than **C** ^**Me**^.^[^ ^46^, ^47^ ^]^ Note that **D** ^**Me**^ plays an important role in the context of the “Wanzlick equilibrium”^[^ ^48^, ^49^ ^]^ since Lemal provided convincing NMR spectroscopic evidence for the uncatalyzed reversible dissociation of **D** ^**Me**^ to afford the corresponding NHC at elevated temperatures in solution;^[^ ^50^ ^]^ the ethyl homologue **D** ^**Et**^ was used for the determination of the dissociation equilibrium constant as a function of temperature.^[^ ^51^ ^]^ Closely related work with the isobutyl congener **D** ^***i*** **Bu**^ was published independently by Hahn.^[^ ^52^ ^]^ The backbone‐unsaturated analogue of **C** ^**Me**^, tetraazafulvalene **E** ^**Me**^, may be viewed as tetraaza analogue of TTF (**A**). **E** ^**Me**^ is expected to be a substantially stronger reductant than **C** ^**Me**^ due to the aromatic character of the imidazolium units formed upon its oxidation to the dication **E** ^**Me**2+^. **E** ^**Me**^ is synthetically accessible, but is very short‐lived due to thermodynamically favorable dissociation affording the corresponding NHC.^[^ ^53^ ^]^ Dissociation is prevented by connecting the N atoms of the molecular halves with (CH_2_)_3_ tethers.^[^ ^54^ ^]^ A value of *E*_1/2_ = −1.20 V versus SCE has been published for this tethered congener **F** in DMF,^[^ ^55^, ^56^ ^]^ making this compound suitable for reductive transformations of organic substrates which had previously required alkali metals or aggressive metal‐containing reductants such as, for example, sodium naphthalenide.^[^ ^57^, ^58^ ^]^

**Figure 2:**
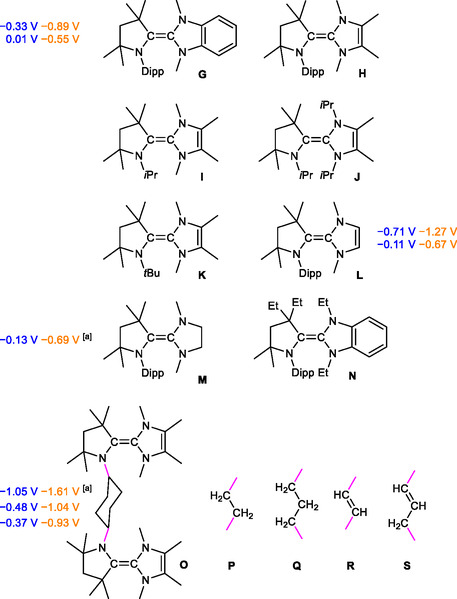
Triaminoalkenes of the NHC–CAAC heterodimer type known to date. *E*_1/2_ values determined by cyclic voltammetry in THF/0.1 m *n*Bu_4_N[PF_6_] are given versus SCE in blue and versus ferrocene/ferrocenium (FcH/FcH^+^) in orange for convenience; for interconversion, *E* ^0^’ (FcH/FcH^+^) = 0.56 V vs SCE in THF/0.1 m *n*Bu_4_N[PF_6_] may be used.^[^ ^67^ ^]^ [a] Two‐electron process.

TTF (**A**) is a much weaker reductant even than TDAE (**B**), which strongly limits its use as synthetic reagent for reductive chemical transformations.^[^ ^57^ ^]^ However, **A** undergoes two well‐separated one‐electron oxidation processes (*E*_1/2_ = 0.19 V (**A** ^0/+^) and 0.58 V (**A** ^+/2+^) vs SCE in DMF; Δ*E*_1/2_ = 0.39 V),^[^ ^46^ ^]^ which is due to the pronounced stability of the intermediate redox state **A** ^+^. It is the stability of this and of closely related cation radicals which is crucial for the widespread use of TTF and its derivatives in materials science. The situation is inverse for tetraazafulvalenes and tetraaminoethenes, which are widely applied in synthesis as strong metal‐free reductants, but are generally not known to give rise to isolable cation radicals offering scope for materials science applications. As the inventor of cyclic (alkyl)(amino)carbenes (CAACs),^[^ ^59^, ^60^, ^61^, ^62^, ^63^, ^64^, ^65^ ^]^ Bertrand realized that triaminoalkenes, which may be viewed as “Wanzlick type” NHC–CAAC heterodimers, are quite different in this context.^[^ ^66^ ^]^ Due to the rather different electronic properties of their two halves, they may exhibit two sequential one‐electron oxidation steps at rather different potentials (**Figure** **2**). The first example, **G**, is the heterodimer of CAAC^Me^, which is the simplest isolable CAAC, and *N*,*N*′‐dimethylbenzimidazolin‐2‐ylidene, which is the NHC produced by dissociation of **D** ^**Me**^. **G** exhibits two well‐separated sequential one‐electron oxidations at *E*_1/2_ = −0.33 V (**G** ^0/+^) and 0.01 V (**G** ^+/2+^) versus SCE in THF (Δ*E*_1/2_ = 0.34 V).^[^ ^46^ ^]^ For comparison, the corresponding values for TTF (**A**) in THF are 0.45 V (**A** ^0/+^) and 0.71 V (**A** ^+/2+^) versus SCE (Δ*E*_1/2_ = 0.26 V).^[^ ^46^, ^68^ ^]^ Remarkably, all three accessible redox states, **G**, **G** ^+^, and **G** ^2+^, were isolated and structurally characterized by X‐ray diffraction (XRD; triflate as counteranion).^[^ ^66^ ^]^ Additional examples (**H**–**S**) were subsequently prepared by Radius,^[^ ^69^ ^]^ Jana,^[^ ^70^, ^71^, ^72^ ^]^ and Munz (Figure 2).^[^ ^46^, ^50^, ^73^ ^]^

**H**, **J**, and **N** have not been studied in terms of their redox behavior.^[^ ^50^, ^69^, ^72^ ^]^ The investigation of **I** by cyclic voltammetry (CV) suffered from poor chemical stability and referencing was not possible; a sequential oxidation with Δ*E*_1/2_ = 0.50 V was observed for **I** at −20 °C.^[^ ^72^ ^]^ Stability issues were less serious for the *t*Bu‐substituted congener **K**, which could be studied at room temperature (Δ*E*_1/2_ = 0.52 V). However, **K** ^+·^ undergoes fast decomposition at room temperature in solution and thus could not be isolated.^[^ ^71^ ^]^ Like **G**, **L** and **M** are both derived from stable CAAC^Me^. Similar to **G**, **L** exhibits two one‐electron oxidation steps at *E*_1/2_ = −1.27 V (**L** ^0/+^) and −0.67 V (**L** ^+/2+^) versus FcH/FcH^+^ in THF/0.1 m *n*Bu_4_N[PF_6_] (Δ*E*_1/2_ = 0.60 V) and gives rise to a cation radical sufficiently stable for isolation; **L** ^+·^(OTf) was structurally characterized by XRD.^[^ ^46^, ^73^ ^]^ In contrast, the electrochemical oxidation of **M** occurs as a single two‐electron step at *E*_1/2_ = −0.69 V (**M** ^0/2+^) under the same conditions.^[^ ^46^ ^]^ Compounds **O**, **P**, and **Q**, which contain two equivalent NHC–CAAC heterodimer moieties connected by a hydrocarbon bridge, react with *n*Bu_4_N[PF_6_] in THF and cyclic voltammetric results could not be obtained.^[^ ^70^ ^]^ However, CV and differential pulse voltammetry (DPV) performed with **O**(OTf)_4_ in acetonitrile/0.1 m *n*Bu_4_N[PF_6_] indicated the formation of the diradical dication **O** ^2+·^by two one‐electron reduction steps at Δ*E*_1/2_ = −0.93 and −1.04 V versus FcH/FcH^+^, followed by a two‐electron step at Δ*E*_1/2_ = −1.61 V furnishing **O** (chemically unstable under these conditions). **O** ^2+·^(OTf)_2_ could be prepared by comproportionation from **O** and **O**(OTf)_4_ and was structurally characterized by XRD. Similar studies revealed that **P** and **Q** do not give rise to persistent diradical dications. No electrochemical data are available for **R** and **S**.^[^ ^70^ ^]^

**Figure 3:**
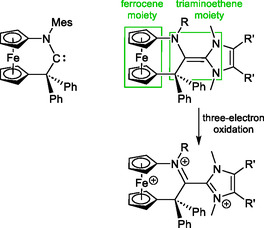
Structure of fcCAAC^Mes^ (left) and of the NHC–fcCAAC heterodimers targeted in the present study (right). Four redox states may be possible for the target compounds, viz., electroneutral, mono‐, di‐, and tricationic (only one resonance structure shown for the latter).

We recently established cyclic (alkyl)(amino)carbenes with a 1,1′‐ferrocenylene backbone (fcCAACs) as an original family by the preparation of the *N*‐mesityl substituted congener [fc(CPh_2_—C—NMes)]^[^ ^74^ ^]^ and have started to address NHC–fcCAAC heterodimers, whose 1,1′‐ferrocenylene unit potentially gives rise to an additional, tricationic, redox state (**Figure** **3**). We here describe first results of this investigation.

## 2 Results and Discussion

### 2.1 Synthesis and Crystal Structures

**Scheme 1:**
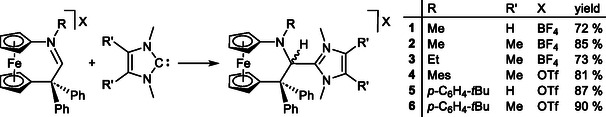
Synthesis of precursors **1**–**6** for NHC–fcCAAC heterodimers (obtained as racemates).

Precursors for NHC–CAAC heterodimers have been shown to be accessible either by reacting a CAAC with a protonated NHC in terms of a C—H insertion or by reacting an NHC with a protonated CAAC in terms of a nucleophilic addition.^[^ ^66^, ^69^, ^70^, ^71^, ^72^, ^73^ ^]^ The former approach is mandatory if the corresponding NHC cannot be isolated, as is the case for the “NHC part” of **M**,^[^ ^46^ ^]^ viz., *N*,*N*′‐dimethylimidazolidin‐2‐ylidene.^[^ ^75^ ^]^ The more common latter approach is mandatory if the corresponding CAAC cannot be isolated, as is the case, inter alia, for the “CAAC part” of **I** or **J**.^[^ ^76^ ^]^ Our previously described fcCAAC precursors gave rise to an isolable CAAC only in the case of the *N*‐mesityl substituted iminium triflate [fc(CPh_2_—CH=NMes)](OTf), whose reaction with KN(SiMe_3_)_2_ (KHMDS) in a mixture of *n*‐hexane and diethyl ether furnished [fc(CPh_2_—C—NMes)] in 68% yield as a crystalline solid. Analogous reactions with the *N*‐alkyl substituted congeners [fc(CPh_2_—CH=NR)][BF_4_] (R = Me, Et) afforded only intractable material.^[^ ^74^ ^]^ The fcCAAC generated from [fc(CPh_2_—CH=N‐*p*‐C_6_H_4_‐*t*Bu)](OTf) and KHMDS in benzene is too short‐lived for isolation due to a fast 1,2‐shift of a phenyl group, furnishing the cyclic enamine [fc{C(Ph)=C(Ph)—N‐*p*‐C_6_H_4_‐*t*Bu}] in almost quantitative yield.^[^ ^77^ ^]^ Together with the fact that the thermal stability of [fc(CPh_2_—CH=NMes)] in solution is limited (*t*_½_ = 4 h at 25 °C) due to an intramolecular insertion of the divalent carbon atom into a methyl C—H bond of the Mes substituent, these results prompted us to choose the nucleophilic addition of an NHC to a protonated CAAC to provide access to precursors for the targeted NHC–fcCAAC heterodimers (**Scheme** **1**).

**Figure 4:**
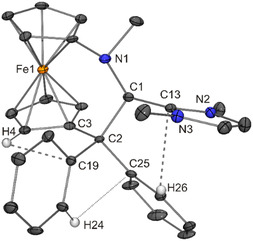
Solid‐state structure of the cation in **1**·CH_2_Cl_2_ (30% probability ellipsoids; H atoms, except those involved in short intramolecular contacts, and solvent molecule omitted for clarity; only one of the two independent molecules is shown). Intramolecular contacts (Å) indicated by dashed or dotted lines: H4···C19 2.63, H24···C25 2.43, H26···C13 2.45.

**Figure 5:**
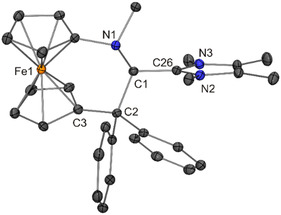
Solid‐state structure of the cation in **2** (30% probability ellipsoids; H atoms omitted for clarity).

**Figure 6:**
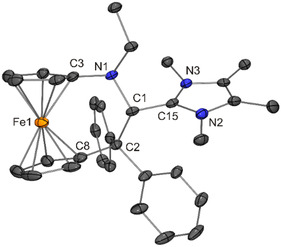
Solid‐state structure of the cation in **3** (30% probability ellipsoids; H atoms omitted for clarity).

**Figure 7:**
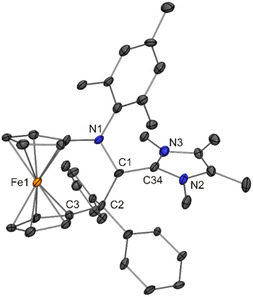
Solid‐state structure of the cation in **4**·Et_2_O (30% probability ellipsoids; H atoms and solvent molecule omitted for clarity).

**Figure 8:**
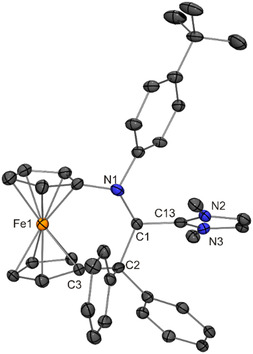
Solid‐state structure of the cation in **5**·2 CH_2_Cl_2_ (30% probability ellipsoids; H atoms and solvent molecules omitted for clarity).

**Figure 9:**
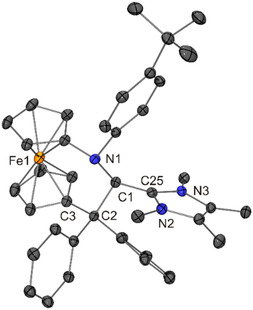
Solid‐state structure of the cation in **6**·CH_2_Cl_2_ (30% probability ellipsoids; H atoms and solvent molecule omitted for clarity).

The permethylated NHC 1,3,4,5‐tetramethylimidazolin‐2‐ylidene (^Me^IMe) was used for each iminium salt. This NHC is readily available and particularly easy to work with due to its excellent crystallinity. *N*,*N*′‐dimethylimidazolin‐2‐ylidene (IMe) was used, too, but in two cases only. Due to its oily nature, it is less convenient from a practical point of view. Compounds **1**–**6** were obtained in good yields (72–90%) by mixing equimolar amounts of the two reactants in toluene at −60 °C, followed by stirring of the respective reaction mixture at room temperature for 17 h. Subsequently, volatile components were removed under vacuum; the remaining yellow solid was washed with diethyl ether and dried under vacuum. Recrystallization was performed by vapor phase diffusion of diethyl ether into a concentrated dichloromethane solution at −20 °C. Regrettably, satisfactory microanalytical data could not be obtained, although recrystallization afforded single crystals suitable for XRD, which allowed the structural characterization of **1**–**6** (**Figure** **4**, **5**, **6**, **7**, **8**, **9**). Pertinent metric parameters are collected in **Table** **1**, which also contains data of the parent iminium salts for comparison.

**Table 1:** Pertinent metric parameters of **1**–**8** (bond lengths in Å, angles in °). Data of the parent [3]ferrocenophane‐type iminium salts are included for comparison.

	C1–N1	C1–C2	C1–C_NHC_	N1–C1–C2	Σ∠N1	Reference
**1** a)	1.486(6) 1.503(5)	1.610(6) 1.606(6)	1.523(6) 1.515(6)	117.1(4) 116.9(4)	333.7 333.8	This work
**2**	1.488(3)	1.608(3)	1.522(3)	118.4(2)	332.5	This work
[fc(CPh_2_—CH= NMe)][BF_4_]	1.284(3)	1.521(3)	–	131.3(2)	360.0	[74]
**3**	1.512(5)	1.619(6)	1.506(7)	118.4(4)	333.4	This work
[fc(CPh_2_—CH=NEt)][BF_4_]	1.292(2)	1.528(2)	–	130.66(15)	360.0	[74]
**4**	1.523(11)	1.653(15)	1.514(14)	117.0(8)	344.8	This work
[fc(CPh_2_—CH=NMes)](OTf)	1.292(3)	1.525(4)	–	130.7(2)	359.8	[74]
**5**	1.486(9)	1.648(9)	1.488(10)	115.1(6)	338.1	This work
**6**	1.489(5)	1.617(5)	1.494(5)	114.7(3)	339.7	This work
[fc(CPh_2_—CH=N‐*p*‐C_6_H_4_‐*t*Bu)](OTf)	1.292(3)	1.513(3)	–	131.7(2)	360.0	[77]
**7**	1.430(4)	1.567(4)	1.375(4)	119.2(3)	355.3	This work
**8**	1.449(3)	1.567(3)	1.390(3)	119.4(2)	359.5	This work

a) Two independent molecules.

The former iminium carbon atom (C1) is a center of chirality. **1**–**6** crystallize as racemic compounds. Despite many attempts, a weakly scattering crystal had to be used in the case of **4** and this crystal structure will not be considered in detail. The lengths of the central C1—C_NHC_ bonds range from 1.49 to 1.52 Å. This is in accord with a C(*sp* ^3^)—C(*sp* ^2^) single bond^[^ ^78^ ^]^ and compares well with closely related compounds, which exhibit bond lengths from 1.48 to 1.53 Å.^[^ ^69^, ^70^, ^72^ ^]^ The C1—N1 bond lengths of **1**–**6** (1.49–1.51 Å) are slightly elongated in comparison to closely related compounds (1.45–1.49 Å).^[^ ^69^, ^70^, ^72^ ^]^ Note that the lengths of C(*sp* ^3^)—N(*sp* ^3^) single bonds in amines typically range from 1.46 to 1.48 Å. A more pronounced elongation is observed for the C1—C2 bonds of **1**–**6**, whose lengths are 1.61–1.65 Å, while closely related compounds exhibit values between 1.56 and 1.59 Å. A structural comparison of **1**–**6** with the respective parent iminium salts reveals a number of characteristic differences (Table 1). Upon nucleophilic addition of the NHC, the iminium C=N double bond (≈1.29 Å) is transformed to a single bond (≈1.50 Å) and the trigonal‐planar iminium nitrogen atom becomes pyramidalized. The tricoordinate iminium carbon atoms exhibit an N1—C1—C2 bond angle of ≈131°. This angle is substantially more acute (by ≈15°) for the tetracoordinate C1 atoms of **1**–**6**. The parent iminium salts have C1—C2 bond lengths from 1.513(3) to 1.528(2) Å,^[^ ^74^, ^77^ ^]^ which is at the upper end of the range typical of C(sp^3^)—C(sp^2^) single bonds (1.49–1.52 Å).^[^ ^42^ ^]^ Notably, the C1—C2 bond lengths of **1**–**6** range from 1.606(6) to 1.648(9) Å (Table 1), which is substantially longer (by up to 0.1 Å) than typical C(*sp* ^3^)—C(*sp* ^3^) single bonds (1.53–1.55 Å).^[^ ^78^ ^]^ The cyclopentadienyl ring tilt angles of **1**–**6** are only moderate (10.8°–13.6°) and very similar to those of the parent iminium salts (11.3°–15.5°). Note that the strained [2]ferrocenophane fc(CMe_2_CMe_2_)^[^ ^79^ ^]^ has a much larger tilt angle of 23.2°, but a substantially shorter C(*sp* ^3^)—C(*sp* ^3^) bridge bond length of only 1.58 Å,^[^ ^80^ ^]^ indicating that the elongated C1—C2 bonds of **1**–**6** are not mainly due to ring strain. We refrain from speculating about possible reasons for this conspicuous structural feature.

The molecular structures of **1**–**6** determined for the solid state by XRD are in concert with NMR spectroscopic data in CD_2_Cl_2_ solution. The ^1^H NMR signal due to the former iminium hydrogen atom is detected as a singlet in the chemical shift range between ≈6.2 and 5.3 ppm, substantially up‐field shifted in comparison with the parent iminium salts (*δ* ≈ 9.8–9.5 ppm). Due to the asymmetric nature of **1**–**6**, the cyclopentadienyl protons are chemically inequivalent and are observed as eight individual NMR signals. In the same vein, the cyclopentadienyl carbon atoms give rise to ten individual signals in the ^13^C NMR spectrum. The ^1^H NMR spectra of **1**–**6** exhibit two conspicuous features, which are addressed in the following. First, one of the cyclopentadienyl ^1^H NMR signals is observed at unusually high field (*δ* ≈ 3.2–2.9 vs 5.1–4.1 ppm for the other cyclopentadienyl signals). Closer inspection of the molecular structures (Figure 1, 4, 5, 6, 7, 9) reveals that the hydrogen atom at one of the carbon atoms flanking the cyclopentadienyl C_ipso_ atom is located above the aromatic π‐system of a phenyl ring, in a position close to the phenyl C_ipso_ atom (CH···C_ipso_ ≈ 2.60 Å; exemplarily indicated for **1** by a dashed line in Figure 4)^[^ ^81^ ^]^ and thus may be experiencing significant shielding due to the aromatic ring current. Second, one of the phenyl hydrogen atoms causes a strongly downfield‐shifted signal (*δ* ≈ 9.9–9.5 vs 7.6–6.5 ppm for the other phenyl signals), except in the case of **4**, where the down‐field shift is less pronounced (*δ* = 8.09 ppm). This suggests a special contact of the phenyl H atom responsible for the downfield‐shifted signal. We note that all compounds listed in Table 1 show a conformational arrangement of their phenyl groups which allows a contact between an *ortho*‐hydrogen atom (H_o_) of one phenyl group and the *ipso*‐carbon atom (C_ipso_) of the other (CH_o_···C_ipso_ 2.43–2.67 Å; exemplarily indicated for **1** by a dotted line in Figure 4), reminiscent of the tilted T‐shaped equilibrium structure of the benzene dimer.^[^ ^82^, ^83^, ^84^, ^85^, ^86^, ^87^, ^88^, ^89^, ^90^, ^91^, ^92^, ^93^, ^94^ ^]^ This contact is also present in the cyclic imine [fc(CPh_2_—CH=N)],^[^ ^74^ ^]^ which is the starting material for the iminium salts listed in Table 1. The phenyl signals of this cyclic imine and the iminium salts derived from it are unexceptional (*δ* ≈ 7.6–7.0 ppm), which indicates that it is not this CH_o_···C_ipso_ contact which is responsible for the downfield shift under discussion. However, in addition to this CH_o_···C_ipso_ contact, **1**–**6** also exhibit a contact between a phenyl *ortho*‐H atom and the cationic formamidinium N_2_C moiety, with CH_o_···C_form_ (exemplarily indicated for **1** by a dashed line in Figure 4) and CH_o_···N_form_ distances between 2.45–2.80 Å and 2.52–2.80 Å,^[^ ^95^ ^]^ respectively. We surmise that it is this additional contact which causes the downfield shift. The H_o_ atom involved in this additional contact belongs to the phenyl ring whose C_ipso_ atom is engaged in the previously discussed CH_o_···C_ipso_ contact. The single exception is **5**. In this case, the same H_o_ atom has a contact to the second phenyl ring as well as to the imidazolium moiety. This bifurcation leads to particularly long distances (CH_o_···C_ipso_ 2.67 Å; CH_o_···C_form_ and CH_o_···N_form_ 2.80 Å).

With compounds **1**–**6** in hand, we investigated their suitability for the synthesis of the target NHC–fcCAAC heterodimers. In contrast to the *N*‐methyl and *N*‐ethyl substituted congeners, the *N*‐mesityl‐substituted fcCAAC [fc(CPh_2_—C—NMes)] is sufficiently stable for isolation.^[^ ^74^ ^]^ This prompted us to start our investigation with the *N*‐mesityl substituted compound **4**. In analogy to the synthetic procedures published for the NHC–CAAC heterodimers shown Figure 2, **4** was treated with KHMDS in toluene. Disappointingly, a mixture was obtained, which could not be separated. Among compounds **1**–**6**, **4** is the sterically most congested congener. Consequently, we next turned our attention to the sterically least congested case, viz., **1**.

**Scheme 2:**
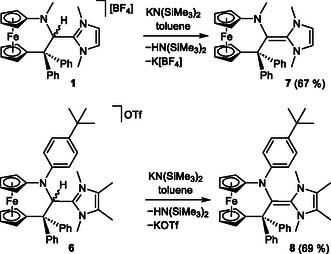
Synthesis of NHC–fcCAAC heterodimers **7** and **8**.

**Figure 10:**
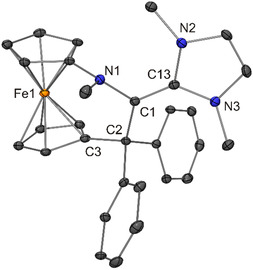
Molecular structure of **7** in the crystal (30% probability ellipsoids; H atoms omitted for clarity).

**Figure 11:**
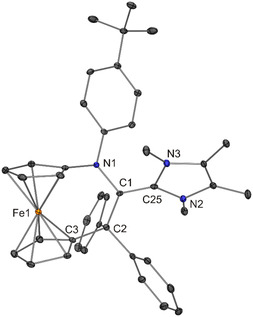
Molecular structure of **8** in the crystal (30% probability ellipsoids; H atoms omitted for clarity).

Gratifyingly, the reaction of **1** with KHMDS in toluene proceeded smoothly and swiftly at room temperature, furnishing the NHC–fcCAAC heterodimer **7** in 67% yield (**Scheme** **2**). In contrast, the analogous reaction of **2** was not successful, affording only intractable material. In view of the pronounced similarity of **1** and **2**, their different behavior is surprising and we cannot offer a plausible explanation for this finding. Finally, in order to prepare an NHC–fcCAAC heterodimer distinctly different from **7**, we selected the *p*‐C_6_H_4_‐*t*Bu substituted compound **6**, expecting that the corresponding NHC–fcCAAC heterodimer **8** would be substantially more electron‐rich than **7**. This expectation was based on the rather different half‐wave potentials published for the tetraminoethenes **C** ^**Ph**^ (−1.28 V) and **C** ^**Me**^ (−0.71 V), the phenyl‐substituted congener being easier to oxidize than the methyl‐substituted one by 0.57 V (Figure 1). On top of that, the permethylated imidazole‐based moiety present in **6** contains twice the number of electron‐donating methyl groups than that of **1**. NHC–fcCAAC heterodimer **8** was isolated in 69% yield (Scheme 2). Triaminoalkenes **7** and **8** were obtained as yellow crystals suitable for XRD. Their molecular structures are shown in **Figure** **10** and **11**.

The ferrocene moiety of **7** is twisted, exhibiting a cg–C_ipso_–C_ipso_–cg (cg = cyclopentadienyl ring centroid) torsion angle of 17.4°, which is in contrast to **8** (torsion angle 0.9°). In both cases, the six‐membered ring formally present in the [3]ferrocenophane‐type unit (composed of the Fe atom, the two cyclopentadienyl C_ipso_ atoms and the three atoms of the NC_2_ bridge) is puckered, adopting an approximate twist boat and half‐chair conformation in the case of **7** and **8**, respectively. The lengths of the central C1—C_NHC_ bonds of **7** and **8** are indistinguishable within experimental error (average value 1.38 Å, Table 1). In line with substantial double‐bond character, this bond is much shorter (by 0.14 and 0.10 Å, respectively) than the corresponding C(*sp* ^3^)—C(*sp* ^2^) single bond of the respective precursor **1** and **6** and lies at the upper end of the range (1.34–1.38 Å) found for the structurally characterized NHC–CAAC heterodimers known to date (**G**,^[^ ^66^ ^]^ **H**,^[^ ^69^ ^]^ **J**,^[^ ^71^ ^]^ **K**,^[^ ^71^ ^]^ **P**–**S** ^[^ ^70^ ^]^). As expected for an alkene, carbon atoms C1 and C_NHC_ of **7** and **8** are in a trigonal‐planar coordination environment (sum of bond angles 360°), in accord with sp^2^ hybridization. However, the triaminoalkene moiety deviates significantly from planarity. The dihedral angle around the C1—C_NHC_ bond is 17.2° for **7** and 32.1° for **8**, reflecting substantially different degrees of steric congestion around the alkene moiety. The value of 32.1° is essentially identical to that reported for the well‐known “twisted” alkene 9,9′‐bifluorenylidene^[^ ^96^, ^97^, ^98^ ^]^ and considerably larger than the angles of **G**, **H**, **J**, **K**, and **P**–**S**, which lie in the range from 19.1° (for **Q**) to 24.3° (for **K**).

The NMR spectra of **7** and **8** in C_6_D_6_ clearly show that the molecular *C*_1_ symmetry observed in the solid state is retained in solution, in accord with a high barrier for planarization of the triaminoalkene moiety and/or the bridge flipping in the [3]ferrocenophane‐type unit. The cyclopentadienyl carbon atoms are chemically inequivalent, giving rise to ten ^13^C NMR signals. Only six signals would be expected for a time‐averaged *C*_s_ symmetric structure. Likewise, eight individual signals are observed for the cyclopentadienyl protons in the ^1^H NMR spectrum of **8**. In the case of **7**, signal overlap leads to two signals integrating for two protons each and four signals integrating for one proton each, whose chemical shift values are not compatible with the highly symmetrical pattern expected for AA′XX′ or AA′BB′ spin systems of a time‐averaged structure with molecular *C*_s_ symmetry. The polarized olefinic double bond present in **7** and **8** is reflected by ^13^C NMR spectroscopic data. The signals due to the carbon atoms C1 and C_NHC_ are observed at *δ*(^13^C) ≈ 100 and 150 ppm, respectively. These chemical shifts are similar to those published for the closely related triaminoalkenes **I**–**S** (Figure 2), which range from 103 to 115 ppm for the signal due to C1 and 140–151 ppm for the signal due to C_NHC_. For further comparison, the corresponding signals of the 1,1‐diaminoethene 1,3,4,5‐tetramethyl‐2‐methyleneimidazoline, which is an extremely polarised olefin,^[^ ^99^, ^100^, ^101^, ^102^, ^103^ ^]^ are located at *δ*(^13^C) ≈ 40 and 154 ppm in C_6_D_6_.^[^ ^104^, ^105^ ^]^

### 2.2 Redox Properties of NHC–fcCAAC Heterodimers 7 and 8

**Figure 12:**
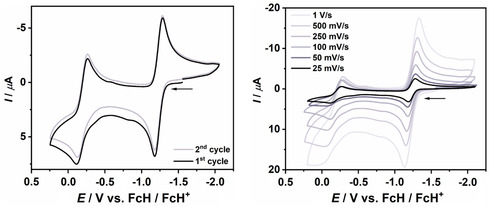
Cyclic voltammogram of **8** in THF with a scan rate of 100 mV s^−1^ (left) and different scan rates (right). *n*Bu_4_N[BAr^F^_4_] supporting electrolyte (0.02 m).

**Figure 13:**
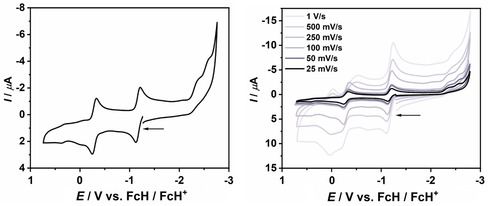
Cyclic voltammogram of **8** in acetonitrile with a scan rate of 100 mV s^−1^ (left) and different scan rates (right). *n*Bu_4_N[BAr^F^_4_] supporting electrolyte (0.02 m).

The redox behavior of NHC–fcCAAC heterodimers **7** and **8** was investigated by CV in THF as well as in acetonitrile with *n*Bu_4_N[BAr^F^_4_] (Ar^F^ = 3,5‐bis(trifluoromethyl)phenyl; 0.02 m) supporting electrolyte. Pertinent electrochemical data are summarized in **Table** **2**. The cyclic voltammograms of **8** in THF and in acetonitrile are shown in **Figure** **12** and **13** and the corresponding ones for **7** are shown in Figure 15 and 16.

**Table 2:** Half‐wave potentials (*E*_1/2_ in V vs FcH/FcH^+^ as pseudo‐reference) of **7** and **8** in THF and acetonitrile solution. Scan rate 100 mV s^−1^. *n*Bu_4_N[BAr^F^_4_] supporting electrolyte (0.02 m). Peak‐to‐peak separation (Δ*E*_p_ in V) given in parentheses.

	First oxidation	Second oxidation	Δ*E*_1/2_
**7** (THF)	−1.13 (0.14)	−0.37 (0.15)	0.76
**7** (MeCN)	−1.11 (0.08)	−0.48 (0.09)	0.63
**8** (THF)	−1.23 (0.11)	−0.19 (0.13)	1.04
**8** (MeCN)	−1.17 (0.09)	−0.29 (0.08)	0.88

**Figure 14:**
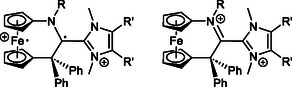
Plausible isomers of the dicationic redox state of NHC–fcCAAC heterodimers. Only one resonance structure is shown in each case.

As described above, NHC–CAAC heterodimers like **G** or **L** undergo two well‐separated one‐electron oxidations (Figure 2). In principle, therefore, an additional, third, one‐electron oxidation may be expected for **7** and **8** due to their redox‐active 1,1′‐ferrocenylene unit. However, only two oxidation steps are observed for both compounds within the potential window of the solvents. Their half‐wave potentials (Table 2) are similar to those of the NHC–CAAC heterodimer **L** (Δ*E*_1/2_ = −1.27 and −0.67 V vs FcH/FcH^+^ in THF/0.1 m *n*Bu_4_N[PF_6_], Figure 2).^[^ ^46^ ^]^ The strongly negative values determined for the first oxidation step of **7** and **8** clearly indicate the electron‐rich nature of these alkenes and point to the site of this oxidation as the triaminoethene unit. Whether this is also the site of the second oxidation is less clear, however, because moderately negative half‐wave potentials down to ≈−0.5 V versus FcH/FcH^+^ are compatible also with an oxidation of the aminoferrocene‐type moiety present in **7** and **8**. For comparison, a half‐wave potential of −0.36 V versus FcH/FcH^+^ has been determined for FcNMe_2_ in acetonitrile.^[^ ^106^ ^]^ In other words, two redox isomers of the dicationic species are plausible (**Figure** **14**).

**Figure 15:**
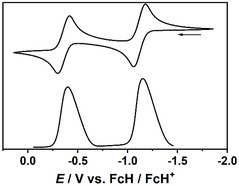
Cyclic voltammogram (top) and differential pulse voltammogram of **7** in THF. Scan rate 25 mV s^−1^. *n*Bu_4_N[BAr^F^_4_] supporting electrolyte (0.02 m).

**Figure 16:**
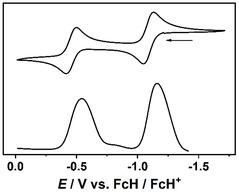
Cyclic voltammogram (top) and differential pulse voltammogram of **7** in acetonitrile. Scan rate 25 mV s^−1^. *n*Bu_4_N[BAr^F^_4_] supporting electrolyte (0.02 m).

Based on the redox potentials alone, it cannot even be excluded that the second oxidation observed for **7** and **8** is a two‐electron process, which involves both the triaminoethene and the ferrocenylene unit, affording the tricationic redox state shown in Figure 3. However, the rather large peak‐to‐peak separations of the redox waves of **7** and **8** point to one‐electron redox processes.^[^ ^107^ ^]^ DPV was performed in order to find out whether the number of electrons transferred in the two consecutive oxidation steps is identical. In the case of **7**, the ratio of the peak area for the two waves determined from the differential pulse voltammogram is close to unity (1.14 and 1.18 in THF and acetonitrile, respectively; **Figure** **15** and **16**), in line with two individual one‐electron processes. **8** showed a similar behavior in acetonitrile (peak area ratio 1.51), while in THF the peak due to the second oxidation almost disappeared during the measurement due to limited stability of the oxidized species in this solvent. In general, a quantification of the DPV data for **8** is difficult due to the rather limited stability of the two‐electron oxidized species for that compound.

**Figure 17:**
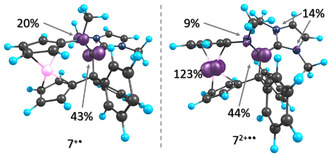
Spin density plot of **7** ^+·^(left, isovalue = 0.025) and **7** ^2+·^(right, isovalue = 0.025).

Quantum‐chemical calculations (PBE0/def2TZVP)^[^ ^108^, ^109^ ^]^ were performed to shed more light on the electronic structure and redox properties of **7** and **8**. The highest occupied molecular orbital (HOMO) and HOMO−1 are, respectively, localized on carbon atom C1 and on the Fe atom in each case. **8** exhibits a significantly higher HOMO energy than **7** (−4.02 vs −4.24 eV), which is in line with the fact that the first oxidation of **8** is significantly easier (by 0.10 V in THF) than that of **7** (Table 2). The computational data indicate that the first oxidation predominantly occurs at carbon atom C1, whereas the second oxidation mainly involves the Fe atom, resulting in a species with a triplet ground state composed, to a first approximation, of a carbon‐centered (spin density ≈45%) and an iron‐centered radical (spin density ≈120%). **Figure** **17** exemplarily shows the calculated spin density plots for **7** ^+·^and **7** ^2+·^ For the two‐electron oxidized species, both the singlet and the triplet states were optimized, and the calculations suggest that for both the compounds the triplet state is substantially stabilized in comparison with the singlet state (Table S12, Supporting Information). The stabilization of the triplet ground state for the two‐electron oxidized species is also likely an explanation for its fleeting nature which has precluded its isolation or characterization.

**Figure 18:**
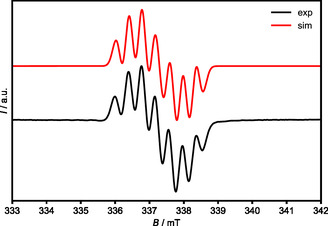
Experimental and simulated (red) EPR spectra of **8** ^+^ in acetonitrile.

The poor stability of these dicationic species in solution prevents an observation of the tricationic redox state by CV. Attempts to generate the dicationic species by chemical oxidation were exemplarily performed with **8**, using two equivalents of silver triflate in THF at −60 °C. A rapid color change from yellow to dark green and concomitant formation of silver metal was observed upon addition of the oxidant. However, only intractable material could be obtained. Use of one equivalent of the oxidant resulted in a color change from yellow to dark brown and concomitant formation of silver metal. In this case work‐up afforded substantial amounts of compound **6**, indicating that cation radical **8** ^+·^is capable of H atom abstraction from the solvent, similar to what Jana has described for **K** ^+·^in THF (Figure 2).^[^ ^71^ ^]^ Nevertheless, it proved possible to characterize **8** ^+·^(generated in situ from **8** and ferrocenium hexafluorophosphate in acetonitrile) by electron paramagnetic resonance (EPR) spectroscopy. The X‐band EPR spectrum (**Figure** **18**) displays a well‐resolved signal centered at *g* = 2.0030. This spectrum was simulated by considering hyperfine coupling of 12.84, 10.42, and 8.76 MHz to three different ^14^N nuclei, close to the values of 14.1, 11.6, and 9.9 MHz reported for **K** ^+·^(generated in situ from **K** and Ag[BF_4_] in THF).^[^ ^71^ ^]^

## 3 Conclusion

We have addressed triaminoalkenes derived from cyclic (alkyl)(amino)carbenes with a 1,1′‐ferrocenylene backbone and NHCs. Such fcCAAC–NHC heterodimers are unprecedented. They proved to be synthetically accessible by nucleophilic addition of stable NHCs to protonated fcCAACs, followed by treatment of the resulting addition product with the strong amide base KHMDS. The target compounds **7** and **8** were obtained in yields close to 70% as analytically pure, crystalline solids by the combination of [fc(CPh_2_—CH=NMe)][BF_4_] with IMe and of [fc(CPh_2_—CH=N‐*p*‐C_6_H_4_‐*t*Bu)](OTf) with ^Me^IMe, respectively. Both compounds are twisted alkenes, the dihedral angle around the triaminoethene double bond C1—C_NHC_ being 17.2° for **7** and 32.1° for **8**. Due to the presence of a triaminoethene unit and a ferrocene moiety, four redox states are expected for such compounds, viz., electroneutral, mono‐, di‐, and tricationic. An investigation of the redox behavior of **7** and **8** by electrochemical methods (CV, DPV) has revealed that these compounds undergo two consecutive one‐electron oxidations, with the poor stability of the dicationic species in solution preventing an observation of the tricationic redox state. A DFT study performed for **7** and **8** shows that the HOMO and HOMO−1 are, respectively, localized on carbon atom C1 of the triaminoethene unit and on the Fe atom in each case. In concert with the EPR spectroscopic analysis of **8** ^+^, computational data indicate that the first oxidation occurs predominantly at carbon atom C1. The second oxidation mainly involves the Fe atom, resulting in a species with a triplet ground state composed, to a first approximation, of a C1‐centered and an Fe‐centered radical.

## 4 Experimental Section

4.1

4.1.1

All reactions involving air‐sensitive compounds were performed in an inert atmosphere (argon or dinitrogen) by using standard Schlenk techniques or a conventional glove box. Starting materials were procured from standard commercial sources and used as received. The NHCs *N*,*N*′‐dimethylimidazolin‐2‐ylidene (IMe)^[^ ^20^ ^]^ and 1,3,4,5‐tetramethylimidazolin‐2‐ylidene (^Me^IMe)^[^ ^110^ ^]^ and the iminium salts [fc(CPh_2_—CH=NR)][BF_4_] (R=Me, Et)^[^ ^74^ ^]^ and [fc(CPh_2_—CH=NR)](OTf) (R = Mes,^[^ ^74^ ^]^ *p*‐C_6_H_4_‐*t*Bu)^[^ ^41^ ^]^ were synthesized according to the reported procedures. NMR spectra were recorded at ambient temperature with Varian NMRS‐500 and MR‐400 spectrometers operating at 500 and 400 MHz, respectively, for ^1^H. Elemental analyses were carried out with a HEKAtech Euro EA‐CHNS elemental analyzer at the Institute of Chemistry, University of Kassel, Germany.

##### Synthesis of 1

[fc(CPh_2_—CH=NMe)][BF_4_] (75 mg, 0.16 mmol) was added to a solution of IMe (15 mg, 0.16 mmol) in toluene (10 mL) cooled to −60 °C. The stirred mixture was allowed to warm up slowly to ambient temperature. After 17 h volatile components were removed under vacuum, affording a light yellow powdery solid, which was washed with diethyl ether (3 × 10 mL) and dried under vacuum. Yield 65 mg (72%). ^1^H NMR (400 MHz, CD_2_Cl_2_): *δ* = 9.61 (d, *J* = 8.2 Hz, 1H, Ph), 7.53 (t, *J* = 7.9 Hz, 1H, Ph), 7.41–7.33 (m, 2H, Ph), 7.32 (d, *J* = 1.9 Hz, 1H, C=CH), 7.29 (t, *J* = 7.2 Hz, 2H, Ph), 7.22–7.09 (m, 3H, Ph), 7.08 (d, *J* = 1.9 Hz, 1H, C=CH), 6.47 (d, *J* = 8.3 Hz, 1H, Ph), 5.30 (s, 1H, Ph_2_CC*H*), 4.80, 4.62, 4.54, 4.47, 4.26, 4.10 (6 m, 6 × 1 H, cyclopentadienyl H), 4.08 (s, 3H, NMe), 4.07 (m, 1H, cyclopentadienyl H), 3.06 (s, 3H, NMe), 2.93 (m, 1H, cyclopentadienyl H), 2.45 ppm (s, 3H, NMe). ^13^C{^1^H} NMR (101 MHz, CD_2_Cl_2_): *δ* = 144.3 (CN_2_), 144.1, 142.6 (2 × Ph C_ipso_), 133.6, 133.3, 130.6, 128.7, 128.4, 126.6 (6 × Ph CH), 124.9, 123.9 (2 × *C*=CH), 105.4, 88.9 (2 × cyclopentadienyl C_ipso_), 77.4 (Ph_2_C*C*H), 75.1, 72.1, 70.8, 69.4 (two closely spaced signals), 68.8, 67.9, 63.4 (8 × cyclopentadienyl CH), 58.7 (Ph_2_ *C*), 46.6, 39.7, 36.3 ppm (3 × NMe). ^19^F NMR (376 MHz, CD_2_Cl_2_): *δ*  = −151.8 ppm.

##### Synthesis of 2

^Me^IMe (23 mg, 0.19 mmol) was added to a suspension of [fc(CPh_2_—CH=NMe)][BF_4_] (89 mg, 0.19 mmol) in toluene (10 mL) cooled to −60 °C. The stirred mixture was allowed to warm up slowly to ambient temperature. After 17 h volatile components were removed under vacuum, affording a yellow solid, which was washed with diethyl ether (3 × 10 mL) and dried under vacuum. Yield 95 mg (85%). ^1^H NMR (400 MHz, CD_2_Cl_2_): *δ* = 9.61 (d, *J* = 8.1 Hz, 1H, Ph), 7.53 (t, *J* = 7.8 Hz, 1H, Ph), 7.35 (m, 2H, Ph), 7.26 (t, *J* = 7.2 Hz, 2H, Ph), 7.11 (m, *J* = 7.8 Hz, 3H, Ph), 6.46 (d, *J* = 8.1 Hz, 1H, Ph), 5.39 (s, 1H, Ph_2_CC*H*), 4.85, 4.72, 4.53, 4.47, 4.26, 4.09, 4.07 (7 m, 7 × 1H, cyclopentadienyl H), 3.95 (s, 3H, NMe), 2.90 (m, 1H, cyclopentadienyl H), 2.87, 2.50 (2 s, 2 × 3 H, NMe), 2.23, 2.03 ppm (2 s, 2 × 3H, C=CMe). ^13^C{^1^H} NMR (101 MHz, CD_2_Cl_2_): *δ* = 144.2 (CN_2_), 143.4, 142.8 (2 × Ph C_ipso_), 133.6, 133.4, 130.9, 128.4, 128.2, 128.0 (6 × Ph CH), 127.6, 126.6 (2 × *C*=CMe), 105.5, 89.0 (2 × cyclopentadienyl C_ipso_,) 77.7 (Ph_2_C*C*H), 75.0, 72.0, 70.7, 69.5, 69.4, 68.7, 67.8, 63.6 (8 × cyclopentadienyl CH), 58.9 (Ph_2_ *C*), 46.6, 35.6, 32.7 (3 × NMe), 9.3, 9.1 ppm (2 × C=*C*Me). ^19^F NMR (376 MHz, CD_2_Cl_2_): *δ* = −152.5 ppm.

##### Synthesis of 3

^Me^IMe (12 mg, 0.10 mmol) was added to a suspension of [fc(CPh_2_—CH=NEt)][BF_4_] (48 mg, 0.10 mmol) in toluene (8 mL) cooled to −60 °C. The stirred mixture was allowed to warm up slowly to ambient temperature. After 17 h volatile components were removed under vacuum, affording a yellow solid, which was washed with diethyl ether (3 × 5 mL) and dried under vacuum. Yield 44 mg (73%). ^1^H NMR (400 MHz, CD_2_Cl_2_): *δ* = 9.63 (d, *J* = 8.1 Hz, 1H, Ph), 7.53 (t, *J* = 7.8 Hz, 2H, Ph), 7.41–7.31 (m, 2H, Ph), 7.25 (t, *J* = 7.4 Hz, 2H, Ph), 7.11 (t, *J* = 7.8 Hz, 2H, Ph), 6.48 (d, *J* = 8.1 Hz, 1H, Ph), 5.49 (s, 1H, Ph_2_CC*H*), 4.88, 4.68 (2 m, 2 × 1 H, cyclopentadienyl H), 4.48 (m, 2H, cyclopentadienyl H), 4.29, 4.11, 4.08 (3 m, 3 × 1H, cyclopentadienyl H), 3.92, 2.90 (2 s, 2 × 3H, NMe), 2.85 (m, 1H, cyclopentadienyl H), 2.55, 2.45 (2 m, 2 × 1H, C*H*_2_CH_3_), 2.23, 2.02 (2 s, 2 × 3 H, C=CMe), 1.02 ppm (t, *J* = 7.0 Hz, 3H, CH_2_C*H*_3_). ^13^C{^1^H} NMR (101 MHz, CD_2_Cl_2_): *δ* = 144.4 (CN_2_), 142.9, 142.8 (2 × Ph C_ipso_), 133.7, 133.3, 130.9, 128.3, 128.1, 127.9 (6 × Ph CH), 127.6, 126.5 (2 × *C*=CMe), 102.4, 89.1 (2 × cyclopentadienyl C_ipso_), 77.3 (Ph_2_C*C*H), 75.0, 72.1, 70.8, 70.7, 69.6, 69.5, 68.1, 63.2 (8 × cyclopentadienyl CH), 59.1 (Ph_2_ *C*), 51.6 (*C*H_2_Me), 35.4, 32.7 (NMe), 14.3 (CH_2_Me), 9.2, 9.1 ppm (2 × C=CMe). ^19^F NMR (376 MHz, CD_2_Cl_2_): *δ* = −152.6 ppm.

##### Synthesis of 4

^Me^IMe (4.8 mg, 0.04 mmol) was added to a suspension of [fc(CPh_2_—CH=NMes)](OTf) (25 mg, 0.04 mmol) in toluene (10 mL) cooled to −60 °C. The stirred mixture was allowed to warm up slowly to ambient temperature. After 17 h volatile components were removed under vacuum, affording a yellow solid, which was washed with diethyl ether (3 × 10 mL) and dried under vacuum. Yield 24 mg (81%). ^1^H NMR (400 MHz, CD_2_Cl_2_): *δ* = 8.09 (m, 1H, Ph), 7.45–7.34 (m, 3H, Ph), 7.31 (t, *J* = 7.3 Hz, 2H, Ph), 7.23 (d, *J* = 7.5 Hz, 1H, Ph), 7.18 (m, 1H, Ph), 6.89 (m, 1H, Ph), 6.81 (m, 1H, Ph), 6.56 (s, 2H, C_6_ *H*_2_Me_3_), 5.67 (s, 1H, Ph_2_CC*H*), 4.97, 4.92 (2 m, 2 × 1 H, cyclopentadienyl H), 4.57 (m, 2H, cyclopentadienyl H), 4.25, 4.2, 4.18 (3 m, 3 × 1H, cyclopentadienyl H), 3.36 (s, 3H, Me), 3.21 (m, 1H, cyclopentadienyl H), 2.77, 2.73, 2.23, 2.12 (4 s, 4 × 3H, Me), 2.00, 1.91 (2 s, 2 × 3H, C=CMe). ^13^C{^1^H} NMR (101 MHz, CD_2_Cl_2_): *δ* = 143.7* (CN_2_), 143.2*, 137.1*, 131.9 (two very closely spaced signals), 131.4, 129.4, 129.0, 128.7, 128.5 (two very closely spaced signals), 127.9, 127.5, (12 × aryl C), 126.0, 124.4 (2 × *C*=CMe), 103.5, 89.4 (2 × cyclopentadienyl C_ipso_), 76.2 (Ph_2_C*C*H), 75.6, 72.6, 71.1, 70.6, 70.1, 69.6, 68.7, 65.0 (8 × cyclopentadienyl CH), 60.0 (Ph_2_ *C*), 35.1, 33.8 (2 × NMe), 22.6, 21.6, 21.5 (3 × mesityl Me), 20.4, 9.4 ppm (2 × C=CMe); CF_3_ not detected (* tentative due to poor signal‐to‐noise ratio (Figure S11, Supporting Information)). ^19^F NMR (376 MHz, CD_2_Cl_2_): *δ* = −78.9 ppm.

##### Synthesis of 5

[fc(CPh_2_—CH=N‐*p*‐C_6_H_4_‐*t*Bu)](OTf) (25 mg, 0.04 mmol) was added to a solution of IMe (3.6 mg, 0.04 mmol) in toluene (10 mL) cooled to −60 °C. The stirred mixture was allowed to warm up slowly to ambient temperature. After 17 h volatile components were removed under vacuum, affording a light yellow powdery solid, which was washed with diethyl ether (3 × 10 mL) and dried under vacuum. Yield 25 mg (87%). ^1^H NMR (400 MHz, CD_2_Cl_2_): *δ* = 9.86 (d, *J* = 10.0 Hz, 1H, Ph), 7.65 (m, 1H, Ph), 7.40 (t, *J* = 7.4 Hz, 1H, Ph), 7.32–7.28 (m, 1H of Ph and 2H of *p*‐C_6_H_4_), 7.24–7.21 (m, 1H of Ph and 2H of *p*‐C_6_H_4_), 7.15 (m, 1H, Ph), 7.09–7.01 (m, 2H of Ph and 1H of C = CH), 6.93 (m, 1H of Ph and 1H of C = CH), 6.51 (d, *J* = 11.4 Hz, 1H, Ph), 6.18 (s, 1H, Ph_2_CCH), 5.04, 4.76, 4.53, 4.51, 4.19, 4.14, 4.07 (7 m, 7 × 1 H, cyclopentadienyl H), 3.74, 3.18 (2 s, 2 × 3 H, NMe), 3.07 (m, 1H, cyclopentadienyl H), 1.19 ppm (s, 9H, *t*Bu). ^13^C{^1^H} NMR (101 MHz, CD_2_Cl_2_): *δ* = 149.8 (CN_2_), 148.8, 144.0, 142.7, 142.3, 133.3, 133.2, 131.7, 128.4, 128.1, 128.0, 127.5, 126.3 (12 × aryl C), 124.0, 122.1 (2 × C = CH), 104.0, 89.2 (2 × cyclopentadienyl C_ipso_), 74.9 (Ph_2_CCH), 74.5, 72.4, 70.9, 69.8 (two very closely spaced signals), 69.7, 68.4, 65.4 (8 × cyclopentadienyl CH), 58.7 (Ph_2_C), 36.0, 35.7 (NMe), 34.8 (CMe_3_), 31.4 ppm (CMe_3_); CF_3_ not detected. ^19^F NMR (376 MHz, CD_2_Cl_2_): *δ* = −78.2 ppm.

##### Synthesis of 6

^Me^IMe (5.8 mg, 0.05 mmol) was added to a suspension of [fc(CPh_2_—CH=N‐*p*‐C_6_H_4_‐*t*Bu)](OTf) (31 mg, 0.05 mmol) in toluene (10 mL) cooled to −60 °C. The stirred mixture was allowed to warm up slowly to ambient temperature. After 17 h volatile components were removed under vacuum, affording a yellow solid, which was washed with diethyl ether (3 × 10 mL) and dried under vacuum. Yield 33 mg (90%). ^1^H NMR (400 MHz, CD_2_Cl_2_): *δ* = 9.84 (d, *J* = 7.9 Hz, 1H, Ph), 7.64 (t, *J* = 7.4 Hz, 1H, Ph), 7.39 (t, *J* = 7.3 Hz, 2H, Ph), 7.29 (t, *J* = 7.2 Hz, 2H, Ph), 7.22–7.20 (m, 1H of Ph and 2H of *p*‐C_6_H_4_), 7.14 (t, *J* = 7.6 Hz, 1H, Ph), 7.02–7.00 (m, 1H of Ph and 2H of *p*‐C_6_H_4_), 6.50 (d, *J* = 7.9 Hz, 1H, Ph), 6.24 (s, 1H, Ph_2_CC*H*), 5.11, 4.76, 4.61, 4.52, 4.21, 4.13, 4.08 (7 m, 7 × 1H, cyclopentadienyl H), 3.55 (s, 3H, NMe), 3.03 (m, 1H, cyclopentadienyl H), 3.01 (s, 3H, NMe), 1.99, 1.88 (2 s, 2 × 3H, C=CMe), 1.19 ppm (s, 9H, *t*Bu). ^13^C{^1^H} NMR (101 MHz, CD_2_Cl_2_): *δ* = 149.6 (CN_2_), 148.1, 144.3, 142.7, 142.4, 133.7, 132.9, 131.3, 128.5, 128.3, 128.2, 127.2, 126.8 (12 × aryl C), 126.8, 124.8 (2 × *C*=CMe), 104.1, 89.4 (2 × cyclopentadienyl C_ipso_), 75.2 (Ph_2_C*C*H), 74.3, 72.4, 70.8, 70.0 (two very closely spaced signals), 69.8, 68.4, 65.3 (8 × cyclopentadienyl CH), 58.9 (Ph_2_ *C*), 35.7 (NMe), 34.7 (*C*Me_3_), 32.7 (NMe), 31.3 (CMe_3_), 9.3, 8.7 ppm (2 × C=CMe); CF_3_ not detected. ^19^F NMR (376 MHz, CD_2_Cl_2_): *δ* = −78.9 ppm.

##### Synthesis of 7

Toluene (3 mL) was added to a mixture of **1** (83 mg, 0.14 mmol) and KHMDS (29 mg, 0.14 mmol). The mixture was stirred for 4 h. Insoluble material was removed by filtration through a short Celite pad. Volatile components were removed from the filtrate. The residue was washed with *n*‐hexane (3 × 2 mL). The light yellow powdery solid was recrystallized from benzene. Yield 47 mg (67%). C_30_H_29_N_3_Fe (487.42): calcd. C 73.92, H 6.00, N 8.62%; found C 73.44, H 6.02, N 8.04%. ^1^H NMR (500 MHz, C_6_D_6_): *δ* = 8.07 (d, *J* = 8.3 Hz, 1H, Ph), 7.64 (d, *J* = 8.7 Hz, 1H, Ph), 7.22 (t, *J* = 7.6 Hz, 1H, Ph), 7.16–6.98 (m, 7H, Ph), 5.57, 5.15 (2 d, *J* = 2.6 Hz, 2 × 1H, C = CH), 4.14 (m, 1H, cyclopentadienyl H), 4.06 (m, 2H, cyclopentadienyl H), 4.02 (m, 1H, cyclopentadienyl H), 3.98 (m, 2H, cyclopentadienyl H), 3.71, 3.65 (2 m, 2 × 1H, cyclopentadienyl H), 3.06, 2.68, 1.99 ppm (3 s, 3 × 3H, NMe). ^13^C{^1^H} NMR (126 MHz, C_6_D_6_): *δ* = 149.6 (NC=CN_2_), 148.7, 147.6 (2 × Ph C_ipso_), 131.4, 128.4, 127.5, 126.5, 126.3, 126.2 (6 × Ph CH), 118.4, 115.9 (2 × *C*=CH), 110.1, 101.3, 96.3 (NC=CN_2_ and 2 × cyclopentadienyl C_ipso_), 74.2, 72.2, 68.5, 67.6, 67.4, 67.1, 65.1, 55.7 (8 × cyclopentadienyl CH), 52.6 (*C*Ph_2_), 43.2, 38.3, 38.0 ppm (3 × NMe).

##### Synthesis of 8

Toluene (2 mL) was added to a mixture of **6** (50 mg, 0.06 mmol) and KHMDS (13 mg, 0.06 mmol). The mixture was stirred for 4 h. Insoluble material was removed by filtration through a short Celite pad. Volatile components were removed from the filtrate. The residue was washed with *n*‐hexane (3 × 2 mL). The light yellow powdery solid was recrystallized from benzene. Yield 28 mg (69%). C_41_H_43_N_3_Fe (633.65): calcd. C 77.72, H 6.84, N 6.63%; found C 77.48, H 7.01, N 6.51%. ^1^H NMR (500 MHz, C_6_D_6_): *δ* = 8.23 (d, *J* = 8.4 Hz, 2H, Ph), 7.25–7.16 (m, 8H, Ph), 7.13–7.10, 7.06–7.03 (2 m, 2 × 2H, *p*‐C_6_H_4_), 4.48, 4.39, 4.33, 4.24, 4.04, 4.00, 3.97, 3.92 (8 m, 8 × 1H, cyclopentadienyl H), 2.76, 2.49 (2 s, 2 × 3H, NMe), 1.23 (s, 3H, C=CMe), 1.22 (s, 9H, *t*Bu), 1.21 ppm (s, 3H, C=CMe). ^13^C{^1^H} NMR (126 MHz, C_6_D_6_): *δ* = 151.1 (NC=*C*N_2_), 147.6, 147.4, 147.1, 139.0, 133.1, 126.8, 126.2, 125.6 (two very closely spaced signals), 125.3 (10 × aryl C), 119.5, 117.4 (2 × *C*=CMe), 116.4 (two very closely spaced signals, 2 × aryl C), 103.0, 100.3, 94.2 (NC=CN_2_ and 2 × cyclopentadienyl C_ipso_), 73.9, 73.4, 71.4, 70.3, 68.8, 68.7, 67.8, 59.8 (8 × cyclopentadienyl CH), 54.5 (*C*Ph_2_), 37.9, 33.9 (2 × NMe), 33.8 (*C*Me_3_), 31.9 (CMe_3_), 9.4, 9.2 ppm (2 × C=CMe).

##### X‐ray Crystallography

For each data collection, a single crystal was mounted on a micromount and all geometric and intensity data were taken from this sample at 100(2) K. Data collections were carried out either on a Stoe IPDS2 diffractometer equipped with a 2‐circle goniometer and an area detector or on a Stoe StadiVari diffractometer equipped with a 4‐circle goniometer and a DECTRIS Pilatus 200 K detector. The data sets were corrected for absorption (by multiscan), Lorentz and polarization effects. The structures were solved by direct methods (SHELXT) and refined using alternating cycles of least‐squares refinements against *F* ^2^ (SHELXL2014/7).^[^ ^111^ ^]^ C‐bonded H atoms were included in the models in calculated positions, heteroatom‐bonded H atoms have been found in the difference Fourier lists. All H atoms were treated with the 1.2‐fold isotropic displacement parameter of their bonding partner. Experimental details for each diffraction experiment are given in Table S1, Supporting Information. CCDC 2415736 (for **1**·CH_2_Cl_2_), 2415737 (for **2**), 2415738 (for **3**), 2415739 (for **4**·Et_2_O), 2415740 (for **5**·2 CH_2_Cl_2_), 2415741 (for **6**·CH_2_Cl_2_), 2415742 (for **7**) and 2415743 (for **8**) contain the supplementary crystallographic data for this article. These data are provided free of charge by the joint Cambridge Crystallographic Data Centre and Fachinformationszentrum Karlsruhe Access Structures service.^[^ ^112^, ^113^, ^114^, ^115^, ^116^, ^117^, ^118^, ^119^, ^120^, ^121^, ^122^, ^123^, ^124^, ^125^, ^126^, ^127^ ^]^

## Supporting Information

The authors have cited additional references cited within the Supporting Information.^[59–66]^ The Supporting Information includes X‐ray crystallographic details, plots of NMR spectra and electrochemical, EPR spectroscopic and computational details.

## Conflict of Interest

The authors declare no conflict of interest.

## Supporting information

Supplementary Material

## Data Availability

The data that support the findings of this study are available in the supplementary material of this article.

## References

[1] J. Broggi , T. Terme , P. Vanelle , Angew. Chem. Int. Ed. 2014, 53, 384. 10.1002/anie.201209060 24273111

[2] K. Deuchert , S. Hünig , Angew. Chem. Int. Ed. 1978, 17, 875.

[3] J. Hocker , R. Merten , Angew. Chem. Int. Ed. Engl. 1972, 11, 964.

[4] R. W. Hoffmann , Angew. Chem. Int. Ed. Engl. 1968, 7, 754.

[5] F. Wudl , G. M. Smith , E. J. Hufnagel , J. Chem. Soc. D 1970, 1453.

[6] D. L. Coffen , J. Q. Chambers , D. R. Williams , P. E. Garrett , N. D. Canfield , J. Am. Chem. Soc. 1971, 93, 2258.

[7] S. Hünig , G. Kießlich , H. Quast , D. Scheutzow , Liebigs Ann. Chem. 1973, 310.

[8] W. R. H. Hurtley , S. Smiles , J. Chem. Soc. 1926, 129, 2263.

[9] F. Wudl , D. Wobschall , E. J. Hufnagel , J. Am. Chem. Soc. 1972, 94, 670.

[10] J. Ferraris , D. O. Cowan , V. Walatka Jr. , J. H. Perlstein , J. Am. Chem. Soc. 1973, 95, 948.

[11] N. Martín , Chem. Commun. 2013, 49, 7025. 10.1039/c3cc00240c 23802195

[12] M. R. Bryce , C. C. Murphy , Nature 1984, 309, 119.

[13] J. H. Perlstein , Angew. Chem. Int. Ed. Engl. 1977, 16, 519.

[14] P. Wu , B. Dharmadhikari , P. Patra , X. Xiong , Nanoscale Adv. 2022, 4, 3418. 36134345 10.1039/d2na00057a PMC9400518

[15] R. Pfattner , S. T. Bromley , C. Rovira , M. Mas‐Torrent , Adv. Funct. Mater. 2016, 26, 2256.

[16] J. J. Bergkamp , S. Decurtins , S.‐X. Liu , Chem. Soc. Rev. 2015, 44, 863. 25256117 10.1039/c4cs00255e

[17] K. P. Goetz , D. Vermeulen , M. E. Payne , C. Kloc , L. E. McNeil , O. A. Jurchescu , J. Mater. Chem. C 2014, 2, 3065.

[18] A. Coskun , J. M. Spruell , G. Barin , W. R. Dichtel , A. H. Flood , Y. Y. Botros , J. F. Stoddard , Chem. Soc. Rev. 2012, 41, 4827. 22648395 10.1039/c2cs35053j

[19] D. Canevet , M. Sallé , G. Zhang , D. Zhang , D. Zhu , Chem. Commun. 2009, 2245. 10.1039/b818607n 19377656

[20] M. Bendikov , F. Wudl , D. F. Perepichka , Chem. Rev. 2004, 104, 4891. 15535637 10.1021/cr030666m

[21] J. L. Segura , N. Martín , Angew. Chem. Int. Ed. 2001, 40, 1372. 10.1002/1521-3773(20010417)40:8<1372::aid-anie1372>3.0.co;2-i 11317287

[22] M. B. Nielsen , C. Lomholt , J. Becher , Chem. Soc. Rev. 2000, 29, 153.

[23] R. L. Pruett , J. T. Barr , K. E. Rapp , C. T. Bahner , J. D. Gibson , R. H. Lafferty Jr. , J. Am. Chem. Soc. 1950, 72, 3646.

[24] K. Kuwata , D. H. Geske , J. Am. Chem. Soc. 1964, 86, 2101.

[25] N. Wiberg , J. W. Buchler , Angew. Chem. Int. Ed. Engl. 1962, 1, 406.

[26] N. Wiberg , Angew. Chem. Int. Ed. Engl. 1968, 7, 766.

[27] M. Kuoboshi , M. Tanaka , S. Kishimoto , K. Goto , M. Mochizuki , H. Tanaka , Tetrahedron Lett. 2000, 41, 81.

[28] C. Burkholder , W. R. Dobier Jr. , M. Médebielle , Tetrahedron Lett. 1997, 38, 821.

[29] M. W. Briscoe , R. D. Chambers , S. J. Mullins , T. Nakamura , J. F. S. Vaughan , F. G. Drakesmith , J. Chem. Soc. Perkin Trans. 1 1994, 3115.

[30] G. Pawelke , J. Fluorine Chem. 1989, 42, 429.

[31] W. Carpenter , J. Org. Chem. 1965, 30, 3082. 14290374

[32] D. J. Charboneau , N. Hazari , H. Huang , M. R. Uehling , S. L. Zultanski , J. Org. Chem. 2022, 87, 7589. 35671350 10.1021/acs.joc.2c00462 PMC9335070

[33] M. Médebielle , W. R. Dolbier Jr. , J. Fluorine Chem. 2008, 129, 930.

[34] P.‐M. Allemand , K. C. Khemani , A. Koch , F. Wudl , K. Holczer , S. Donovan , G. Grüner , J. D. Thompson , Science 1991, 253, 301. 17794696 10.1126/science.253.5017.301

[35] A. J. Arduengo III , R. L. Harlow , M. Kline , J. Am. Chem. Soc. 1991, 113, 361.

[36] P. Bellotti , M. Koy , M. N. Hopkinson , F. Glorius , Nat. Rev. Chem. 2021, 5, 711. 37118184 10.1038/s41570-021-00321-1

[37] M. N. Hopkinson , C. Richter , M. Schedler , F. Glorius , Nature 2014, 510, 485. 24965649 10.1038/nature13384

[38] H.‐W. Wanzlick , H.‐J. Kleiner , Angew. Chem. 1961, 73, 493.

[39] H.‐W. Wanzlick , E. Schikora , Chem. Ber. 1961, 94, 2389.

[40] H.‐W. Wanzlick , E. Schikora , Angew. Chem. 1960, 72, 494.

[41] D. M. Lemal , K. I. Kawano , J. Am. Chem. Soc. 1962, 84, 1761.

[42] H. E. Winberg , J. E. Carnahan , D. D. Coffman , M. Brown , J. Am. Chem. Soc. 1965, 87, 2055.

[43] H.‐W. Wanzlick , F. Esser , H.‐J. Kleiner , Chem. Ber. 1963, 96, 1208.

[44] C. Burkholder , W. R. Dolbier Jr. , M. Médebielle , J. Org. Chem. 1998, 63, 5385.

[45] H. Bock , D. Jaculi , Angew. Chem. Int. Ed. Engl. 1984, 23, 305.

[46] J. Messelberger , A. Grünwald , S. J. Goodner , F. Zeilinger , P. Pinter , M. E. Miehlich , F. W. Heinemann , M. M. Hansmann , D. Munz , Chem. Sci. 2020, 11, 4138. 34760147 10.1039/d0sc00699h PMC8562513

[47] S. Hünig , H. Schlaf , G. Kießlich , D. Scheutzow , Tetrahedron Lett. 1969, 27, 2271.

[48] R. W. Alder , M. E. Blake , L. Chaker , J. N. Harvey , F. Paolini , J. Schütz , Angew. Chem. Int. Ed. 2004, 43, 5896. 10.1002/anie.200400654 15457494

[49] V. P. W. Böhm , W. A. Herrmann , Angew. Chem. Int. Ed. 2000, 39, 4036. 10.1002/1521-3773(20001117)39:22<4036::aid-anie4036>3.0.co;2-l 11093196

[50] J. Messelberger , M. Kumar , S. J. Goodner , D. Munz , Org. Chem. Front. 2021, 8, 6663.

[51] Y. Liu , P. E. Lindner , D. M. Lemal , J. Am. Chem. Soc. 1999, 121, 10626.

[52] F. E. Hahn , L. Wittenbecher , D. Le Van , R. Fröhlich , Angew. Chem. Int. Ed. 2000, 39, 541. 10.1002/(sici)1521-3773(20000204)39:3<541::aid-anie541>3.0.co;2-b 10671250

[53] P. I. Jolly , S. Zhou , D. W. Thompson , J. Garnier , J. A. Parkinson , T. Tuttle , J. A. Murphy , Chem. Sci. 2012, 3, 1675.

[54] T. A. Taton , P. Chen , Angew. Chem. Int. Ed. Engl. 1996, 35, 1011.

[55] J. R. Ames , M. A. Houghtaling , D. L. Terrian , T. P. Mitchell , Can. J. Chem. 1997, 75, 28.

[56] J. A. Murphy , J. Garnier , S. R. Park , F. Schoenebeck , S. Zhou , A. T. Turner , Org. Lett. 2008, 10, 1227. 18288858 10.1021/ol800134g

[57] E. Doni , J. A. Murphy , Chem. Commun. 2014, 50, 6073. 10.1039/c3cc48969h 24690952

[58] R. D. Richardson , T. Wirth , Chem. Unserer Zeit 2008, 42, 186.

[59] V. Lavallo , Y. Canac , C. Präsang , B. Donnadieu , G. Bertrand , Angew. Chem. Int. Ed. 2005, 44, 5705. 10.1002/anie.200501841 PMC2427276 16059961

[60] S. Kumar Kushvaha , A. Mishra , H. W. Roesky , K. Chandra Mondal , Chem. Asian J. 2022, 17, e202101301. 34989475 10.1002/asia.202101301 PMC9307053

[61] M. Melaimi , R. Jazzar , M. Soleilhavoup , G. Bertrand , Angew. Chem. Int. Ed. 2017, 56, 10046. 10.1002/anie.201702148 28376253

[62] U. S. D. Paul , U. Radius , Eur. J. Inorg. Chem. 2017, 3362.

[63] S. Roy , K. Chandra Mondal , H. W. Roesky , Acc. Chem. Res. 2016, 49, 357. 26925983 10.1021/acs.accounts.5b00381

[64] M. Soleilhavoup , G. Bertrand , Acc. Chem. Res. 2015, 48, 256. 25515548 10.1021/ar5003494

[65] M. Melaimi , M. Soleilhavoup , G. Bertrand , Angew. Chem. Int. Ed. 2010, 49, 8810. 10.1002/anie.201000165 PMC3130005 20836099

[66] D. Munz , J. Chu , M. Melaimi , G. Bertrand , Angew. Chem. Int. Ed. 2016, 55, 12886. 10.1002/anie.201607537 27628756

[67] N. G. Connelly , W. E. Geiger , Chem. Rev. 1996, 96, 877. 11848774 10.1021/cr940053x

[68] D. L. Lichtenberger , R. L. Johnston , K. Hinkelmann , T. Suzuki , F. Wudl , J. Am. Chem. Soc. 1990, 112, 3302.

[69] U. S. D. Paul , U. Radius , Chem. Eur. J. 2017, 23, 3993. 28139870 10.1002/chem.201605950

[70] M. K. Nayak , P. Sarkar , B. J. Elvers , S. Mehta , F. Zhang , N. Chrysokos , I. Krummenacher , T. Vijayakanth , R. S. Narayanan , R. Dolai , B. Roy , V. Malik , H. Rawat , A. Mondal , R. Boomishankar , S. K. Pati , H. Braunschweig , C. Schulzke , P. Ravat , A. Jana , Chem. Sci. 2022, 13, 12533. 36382295 10.1039/d2sc03937k PMC9629079

[71] D. Mandal , R. Dolai , R. Kumar , S. Suhr , N. Chrysokos , P. Kalita , R. S. Narayanan , G. Rajaraman , C. Schulzke , B. Sarkar , V. Chandrasekhar , A. Jana , J. Org. Chem. 2019, 84, 8899. 31187990 10.1021/acs.joc.9b00774

[72] D. Mandal , R. Dolai , N. Chrysokos , P. Kalita , R. Kumar , D. Dhara , A. Maiti , R. S. Narayanan , G. Rajaraman , C. Schulzke , V. Chandrasekhar , A. Jana , Org. Lett. 2017, 19, 5605. 28968127 10.1021/acs.orglett.7b02721

[73] A. Grünwald , S. J. Goodner , D. Munz , J. Vis. Exp. 2019, 146, e59389. 10.3791/59389 31058905

[74] J. Volk , M. Heinz , M. Leibold , C. Bruhn , T. Bens , B. Sarkar , M. C. Holthausen , U. Siemeling , Chem. Commun. 2022, 58, 10396. 10.1039/d2cc03871d 36039867

[75] M. K. Denk , A. Thadani , K. Hatano , A. Lough , Angew. Chem. Int. Ed. Engl. 1997, 36, 2607.

[76] D. Mandal , R. Dolai , P. Kalita , R. S. Narayanan , R. Kumar , S. Sobottka , B. Sarkar , G. Rajaraman , V. Chandrasekhar , A. Jana , Chem. Eur. J. 2018, 24, 12722. 29797625 10.1002/chem.201802587

[77] J. Volk , M. Heinz , R. Guthardt , S. Yadav , C. Bruhn , M. C. Holthausen , U. Siemeling , Chem. Eur. J. 2024, 30, e202403028. 39225629 10.1002/chem.202403028

[78] E. V. Anslyn , D. A. Dougherty , Modern Physical Organic Chemistry, University Science Books, Sausalito, CA 2004, p. 22.

[79] J. M. Nelson , H. Rengel , I. Manners , J. Am. Chem. Soc. 1993, 115, 7035.

[80] M. B. Laing , K. N. Trueblood , Acta Crystallogr. 1965, 19, 373.

[81] S. Alvarez , Dalton Trans. 2013, 42, 8617. 23632803 10.1039/c3dt50599e

[82] A. K. Tummanapelli , S. Vasudevan , J. Chem. Phys. 2014, 140, 227102. 24929417 10.1063/1.4882016

[83] A. van der Avoird , R. Podeszwa , B. Ensing , K. Szalewicz , J. Chem. Phys. 2014, 140, 227101. 24929416 10.1063/1.4882015

[84] A. K. Tummanapelli , S. Vasudevan , J. Chem. Phys. 2013, 139, 201102. 24289335 10.1063/1.4834855

[85] M. Schnell , U. Erlekam , P. R. Bunker , G. von Helden , J.‐U. Grabow , G. Meijer , A. van der Avoird , Angew. Chem. Int. Ed. 2013, 52, 5180. 10.1002/anie.201300653 23589451

[86] O. Bludský , M. Rubeš , P. Soldán , P. Nachtigall , J. Chem. Phys. 2008, 128, 114102. 18361549 10.1063/1.2890968

[87] M. Pitoňák , P. Neogrády , J. Řezáč , P. Jurečka , M. Urban , P. Hobza , J. Chem. Theory Comput. 2008, 4, 1829. 26620326 10.1021/ct800229h

[88] R. Podeszwa , R. Bukowski , K. Szalewicz , J. Phys. Chem. A 2006, 110, 10345. 16928128 10.1021/jp064095o

[89] J. A. Frey , C. Holzer , W. Klopper , S. Leutwyler , Chem. Rev. 2016, 116, 5614. 27055105 10.1021/acs.chemrev.5b00652

[90] A. R. Gadre , S. D. Yeole , N. Sahu , Chem. Rev. 2014, 114, 12132. 25341561 10.1021/cr4006632

[91] J. D. Dunitz , A. Gavezzotti , Angew. Chem. Int. Ed. 2005, 44, 1766. 10.1002/anie.200460157 15685679

[92] H. Brunner , G. Balász , T. Tsuno , H. Iwabe , ACS Omega 2018, 3, 982. 31457942 10.1021/acsomega.7b01460 PMC6641508

[93] H. Brunner , T. Tsuno , Dalton Trans. 2017, 46, 5103. 28345087 10.1039/c7dt00474e

[94] H. Brunner , T. Tsuno , Inorg. Chim. Acta 2016, 446, 132.

[95] The sum of the van der Waals radii of H (1.20 Å) and N (1.66 Å) is 2.86 Å; see ref. [81].

[96] J. S. Lee , S. C. Nyburg , Acta Crystallogr. C: Cryst. Struct. Commun. 1985, 41, 560.

[97] G. L. Eakins , M. W. Cooper , N. N. Gerasimchuk , T. J. Phillips , B. E. Breyfogle , C. J. Stearman , Can. J. Chem. 2013, 91, 1059.

[98] D. Lenoir , P. J. Smith , J. F. Liebman , Strained Hydrocarbons (Ed: H. Doziuk ), Wiley‐VCH, Weinheim 2009, pp. 103–112.

[99] A. Doddi , M. Peters , M. Tamm , Chem. Rev. 2019, 119, 6994. 30983327 10.1021/acs.chemrev.8b00791

[100] S. Naumann , Chem. Commun. 2019, 55, 11658. 10.1039/c9cc06316a 31517349

[101] M. M. D. Roy , E. Rivard , Acc. Chem. Res. 2017, 50, 2017. 28777537 10.1021/acs.accounts.7b00264

[102] R. C. Crocker , T. V. Nguyen , Chem. Eur. J. 2016, 22, 2208.26608598

[103] R. S. Ghadwal , Dalton Trans. 2016, 45, 16081. 27427200 10.1039/c6dt02158a

[104] R. Guthardt , C. Bruhn , U. Siemeling , Polyhedron 2021, 194, 114959.

[105] N. Kuhn , H. Bohnen , J. Kreutzberg , D. Bläser , R. Boese , J. Chem. Soc., Chem. Commun. 1993, 1136.

[106] W. E. Britton , R. Kashyap , M. El‐Hashash , M. El‐Kady , M. Herberhold , Organometallics 1986, 5, 1029.

[107] P. Zanello , Inorganic Electrochemistry: Theory, Practice and Application, RSC Cambridge, UK 2003, p. 56.

[108] F. Weigend , R. Ahlrichs , Phys. Chem. Chem. Phys. 2005, 7, 3297. 16240044 10.1039/b508541a

[109] C. Adamo , V. Barone , J. Chem. Phys. 1999, 110, 6158.

[110] N. Kuhn , T. Kratz , Synthesis 1993, 561.

[111] G. M. Sheldrick , Acta Crystallogr. Sect. A 2008, 64, 112.18156677

[112] F. Neese , Wiley Interdiscip. Rev.: Comput. Mol. Sci. 2012, 2, 73.

[113] F. Neese , Wiley Interdiscip. Rev.: Comput. Mol. Sci. 2022, 12, e1606.

[114] A. V. Marenich , C. J. Cramer , D. G. Truhlar , J. Phys. Chem. B 2009, 113, 6378. 19366259 10.1021/jp810292n

[115] V. Barone , M. Cossi , J. Phys. Chem. A 1998, 102, 1995.

[116] P.‐O. Löwdin , J. Appl. Phys. 1962, 33, 251.

[117] T. Petrenko , S. Kossmann , F. Neese , J. Chem. Phys. 2011, 134, 054116. 21303101 10.1063/1.3533441

[118] F. Neese , G. Olbrich , Chem. Phys. Lett. 2002, 362, 170.

[119] R. Izsák , F. Neese , J. Chem. Phys. 2011, 135, 144105. 22010696 10.1063/1.3646921

[120] J. L. Whitten , J. Chem. Phys. 1973, 58, 4496.

[121] O. Vahtras , J. Almlöf , M. W. Feyereisen , Chem. Phys. Lett. 1993, 213, 514.

[122] F. Neese , F. Wennmohs , A. Hansen , U. Becker , Chem. Phys. 2009, 356, 98.

[123] K. Eichkorn , O. Treutler , H. Öhm , M. Häser , R. Ahlrichs , Chem. Phys. Lett. 1995, 242, 652.

[124] K. Eichkorn , F. Weigend , O. Treutler , R. Ahlrichs , Theor. Chem. Acc. 1997, 97, 119.

[125] F. Weigend , Phys. Chem. Chem. Phys. 2006, 8, 1057. 16633586 10.1039/b515623h

[126] S. Grimme , J. Antony , S. Ehrlich , H. Krieg , J. Chem. Phys. 2010, 132, 154104. 20423165 10.1063/1.3382344

[127] ChemCraft , *Graphical Software for Visualisation of Quantum Chemistry Computations*, https://www.chemcraftprog.com

