# Supplementary material for: Triaminoalkenes Derived from Cyclic (Alkyl)(amino)carbenes with a 1,1′‐Ferrocenylene Backbone and N‐Heterocyclic Carbenes: fcCAAC–NHC Heterodimers
Source: ChemistryOpen. 2025 Apr 14;14(10):e202500156. doi: 10.1002/open.202500156 (PMC12518052; doi:10.1002/open.202500156)
Supplement: Supplementary file 1 — Supplementary Material [file OPEN-14-e202500156-s001.pdf]

Table of Contents

**A**	**X-Ray Crystallography**	S2
**B**	**Plots of NMR Spectra**	S3
**C**	**Electrochemistry**	S14
**D**	**Electron Paramagnetic Resonance Spectroscopy**	S14
**E**	**DFT Calculations**	S14
**F**	**References**	S27

A X-Ray Crystallography

Table S1. X-ray crystallographic details.

	**1**·CH_2Cl_2	**2**	**3**	**4**·Et_2O	**5**·2 CH_2Cl_2	**6**·CH_2Cl_2	**7**	**8**
Empirical formula	$C_{31}H_{32}BCl_2F_4FeN_3$	$C_{32}H_{34}BF_4FeN_3$	$C_{33}H_{36}BF_4FeN_3$	$C_{45}H_{52}F_3FeN_3O_4S$	$C_{42}H_{44}Cl_4F_3FeN_3O_3S$	$C_{43}H_{46}Cl_2F_3FeN_3O_3S$	$C_{30}H_{29}FeN_3$	$C_{41}H_{43}FeN_3$
Formula weight	660.15	603.28	617.31	843.80	925.51	868.64	487.41	633.63
Crystal system	triclinic	monoclinic	monoclinic	monoclinic	monoclinic	monoclinic	monoclinic	monoclinic
Space group	*P*-1	$P2_1/n$	$P2_1/n$	$P2_1/c$	$C2/c$	$P2_1/n$	$P2_1/n$	$P2_1/c$
a/Å	11.0078(10)	9.3678(15)	9.5221(4)	11.279(2)	39.48(2)	14.431(2)	13.4943(7)	16.1589(7)
b/Å	13.4744(12)	22.211(5)	22.1641(11)	19.261(3)	9.226(3)	15.2400(15)	11.5572(5)	10.9920(4)
c/Å	20.3873(19)	13.3706(17)	13.4922(6)	18.528(5)	25.671(12)	18.793(5)	14.5614(9)	18.5347(9)
α/°	100.735(7)	90	90	90	90	90	90	90
β/°	101.477(8)	93.886(12)	91.066(4)	93.652(17)	113.18(4)	102.887(17)	96.846(4)	100.722(4)
γ/°	89.902(7)	90	90	90	90	90	90	90
Volume/Å^3	2909.6(5)	2775.6(8)	2847.0(2)	4017.0(13)	8596(7)	4029.2(12)	2254.8(2)	3234.6(2)
Z	4	4	4	4	8	4	4	4
ρ_{calcd}/gcm^{-3}	1.507	1.444	1.440	1.395	1.430	1.432	1.436	1.301
μ/mm^{-1}	6.302	4.821	4.713	4.011	6.017	0.617	0.694	3.986
F(000)	1360.0	1256.0	1288.0	1776.0	3824.0	1808.0	1024.0	1344.0
Crystal size/mm^3	0.14 × 0.093 × 0.06	0.41 × 0.22 × 0.12	0.23 × 0.10 × 0.02	0.17 × 0.12 × 0.06	0.35 × 0.15 × 0.02	0.28 × 0.13 × 0.04	0.35 × 0.17 × 0.03	0.10 × 0.08 × 0.05
Radiation used	Cu Kα (λ = 1.54186 Å)	Cu Kα (λ = 1.54186 Å)	Cu Kα (λ = 1.54186 Å)	Cu Kα (λ = 1.54186 Å)	Cu Kα (λ = 1.54186 Å)	Mo Kα (λ = 0.71073 Å)	Mo Kα (λ = 0.71073 Å)	Cu Kα (λ = 1.54186 Å)
2Θ range/°	6.682 to 141.492	7.73 to 141.268	7.672 to 141.278	7.854 to 137.96	7.15 to 138.996	3.234 to 51.85	3.89 to 51.868	9.398 to 142.164
	–6 ≤ h ≤ 13	–10 ≤ h ≤ 6	–11 ≤ h ≤ 5	–9 ≤ h ≤ 13	–47 ≤ h ≤ 45	–17 ≤ h ≤ 16	–16 ≤ h ≤ 16	–19 ≤ h ≤ 19
Index ranges	–16 ≤ k ≤ 15	–26 ≤ k ≤ 26	–26 ≤ k ≤ 23	–23 ≤ k ≤ 12	–11 ≤ k ≤ 6	–18 ≤ k ≤ 16	–14 ≤ k ≤ 13	–5 ≤ k ≤ 13
	–24 ≤ l ≤ 24	–15 ≤ l ≤ 16	–14 ≤ l ≤ 16	–21 ≤ l ≤ 22	–25 ≤ l ≤ 31	–23 ≤ l ≤ 22	–17 ≤ l ≤ 17	–22 ≤ l ≤ 19
Reflections collected	25818	17168	13654	19343	17807	16538	11799	14359
Independent reflections	10777 [R_{int} = 0.0390]	5159 [R_{int} = 0.0431]	5217 [R_{int} = 0.0758]	7380 [R_{int} = 0.1625]	7859 [R_{int} = 0.0940]	7659 [R_{int} = 0.0483]	4325 [R_{int} = 0.0451]	6076 [R_{int} = 0.0328]
Data/restraints/parameters	10777/0/763	5159/0/375	5217/0/420	7380/0/523	7859/0/519	7659/0/512	4325/0/310	6076/0/413
Goodness-of-fit on F^2	1.098	1.071	1.018	1.043	1.017	1.031	1.060	1.031
Final *R* indexes	*R*1 = 0.0724	*R*1 = 0.0520	*R*1 = 0.0716	*R*1 = 0.1169	*R*1 = 0.0891	*R*1 = 0.0619	*R*1 = 0.0506	*R*1 = 0.0425
[$I > 2\sigma(I)$]	*wR*2 = 0.1908	*wR*2 = 0.1399	*wR*2 = 0.1706	*wR*2 = 0.2632	*wR*2 = 0.2187	*wR*2 = 0.1576	*wR*2 = 0.1015	*wR*2 = 0.0841
Final *R* indexes	*R*1 = 0.0848	*R*1 = 0.0558	*R*1 = 0.1192	*R*1 = 0.2328	*R*1 = 0.1543	*R*1 = 0.0929	*R*1 = 0.0745	*R*1 = 0.0661
[all data]	*wR*2 = 0.1986	*wR*2 = 0.1445	*wR*2 = 0.2026	*wR*2 = 0.3571	*wR*2 = 0.2789	*wR*2 = 0.1776	*wR*2 = 0.1136	*wR*2 = 0.0926
Largest diff. peak/hole/eÅ^{-3}	1.28/–0.70	0.60/–0.57	0.55/–0.46	1.21/–0.74	0.79/–0.89	1.05/–0.86	0.56/–0.46	0.34/–0.25
CCDC No.	2415736	2415737	2415738	2415739	2415740	2415741	2415742	2415743

B Plots of NMR Spectra

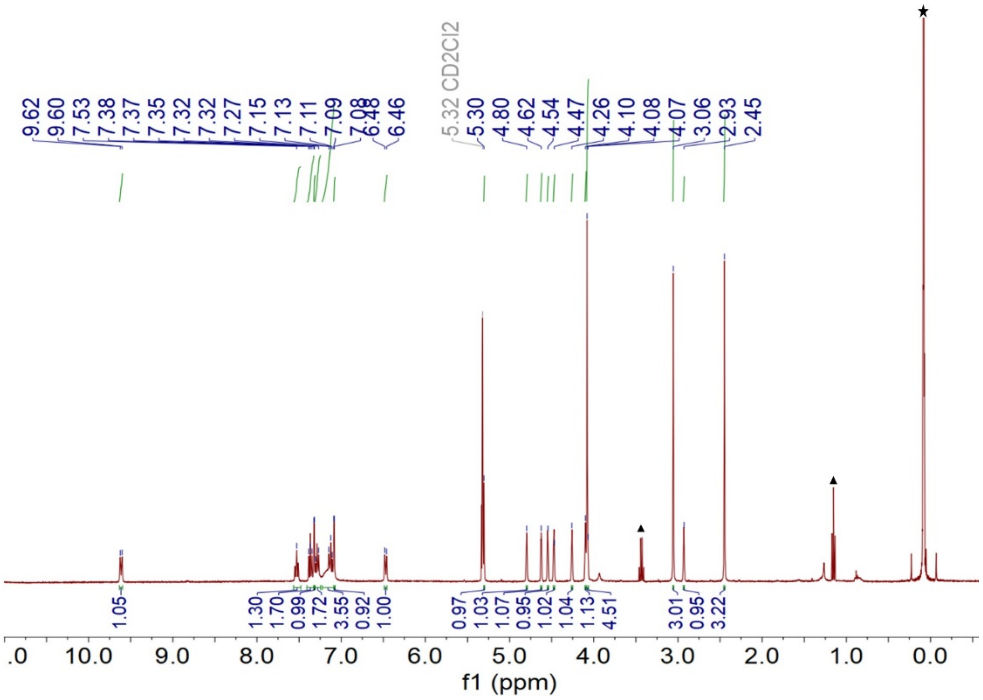

Figure S1. ^{1}H NMR spectrum (400 MHz, CD_2Cl_2) of **1**. The signals marked belong to silicon grease (*) and diethyl ether (▲).

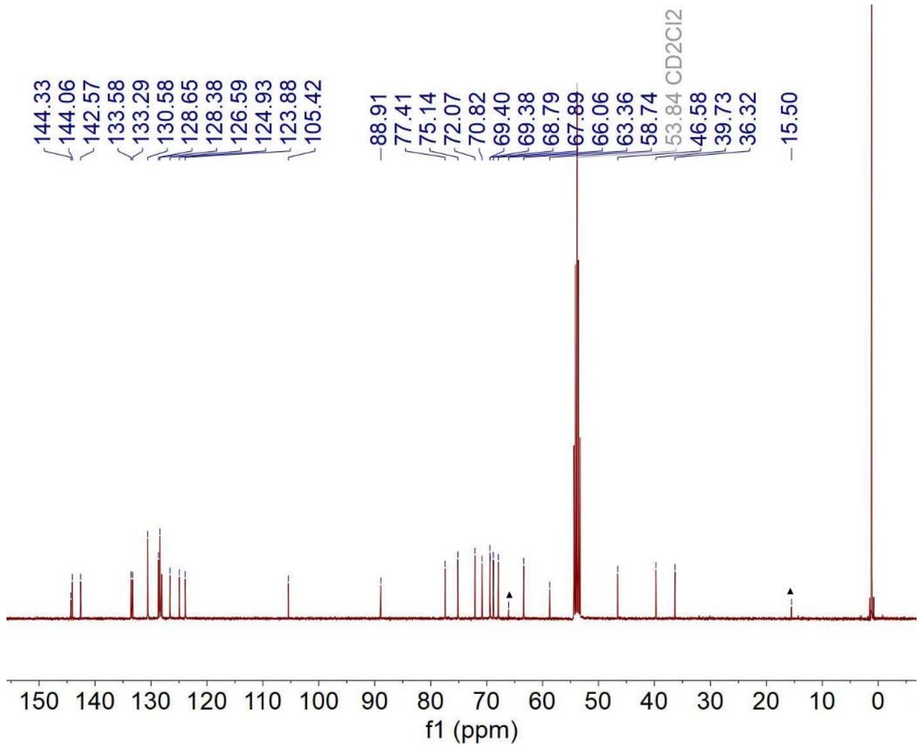

Figure S2. $^{13}C\{^{1}H\}$ NMR spectrum (101 MHz, CD_2Cl_2) of **1**. The signals marked belong to diethyl ether (▲).

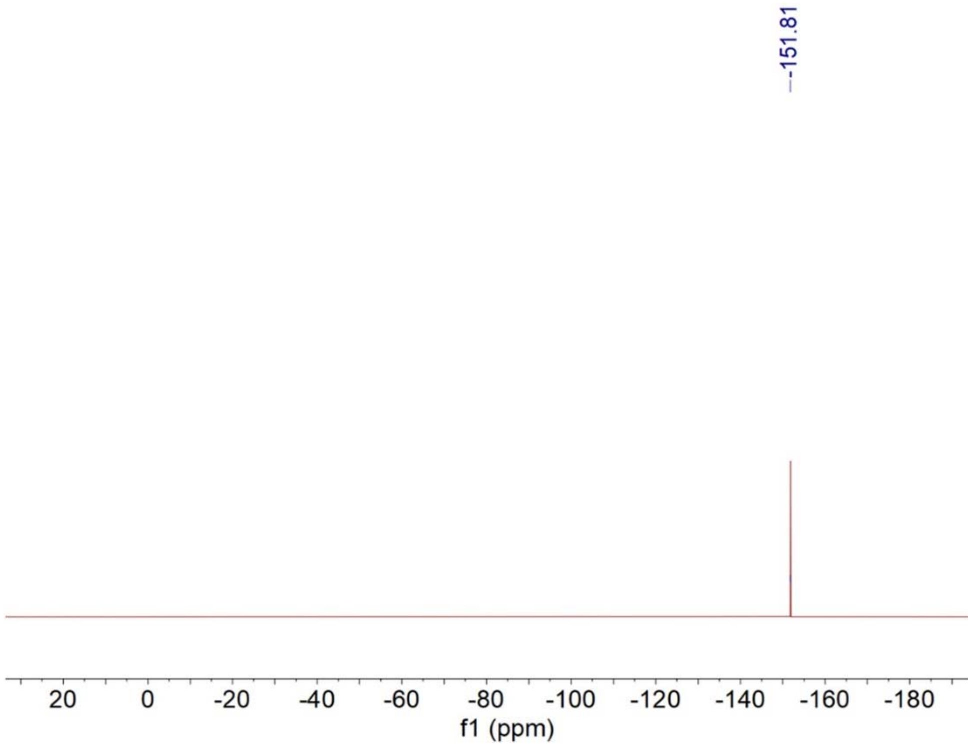

Figure S3. ^{19}F NMR spectrum (376 MHz, CD_2Cl_2) of **1**.

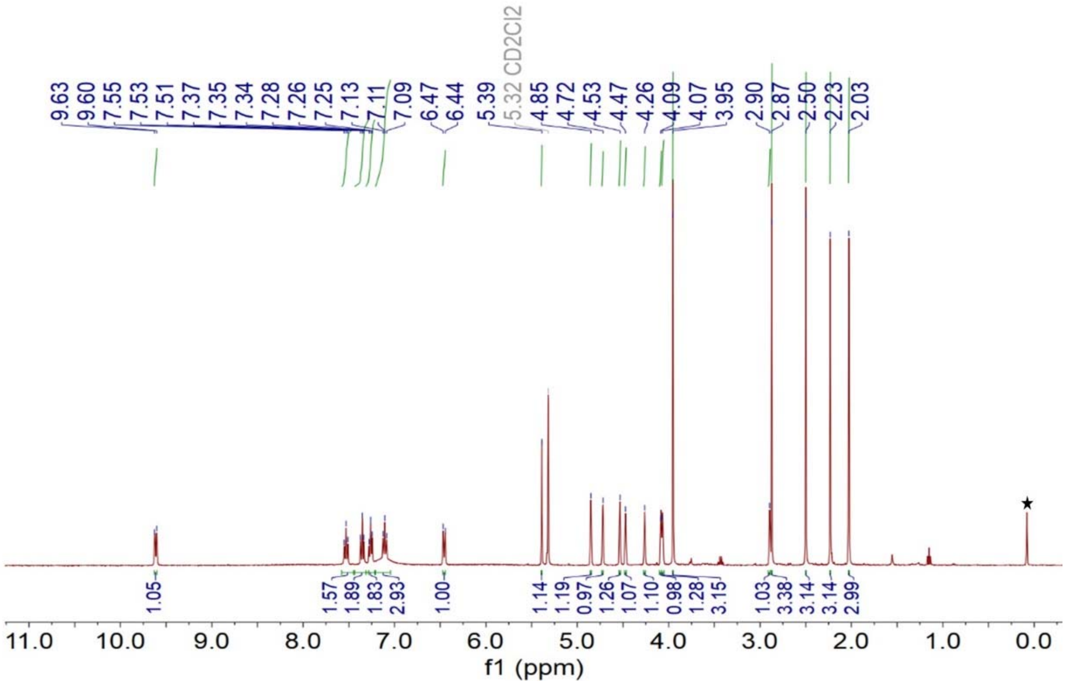

Figure S4. ^{1}H NMR spectrum (400 MHz, CD_2Cl_2) of **2**. The signal marked belongs to silicon grease (*).

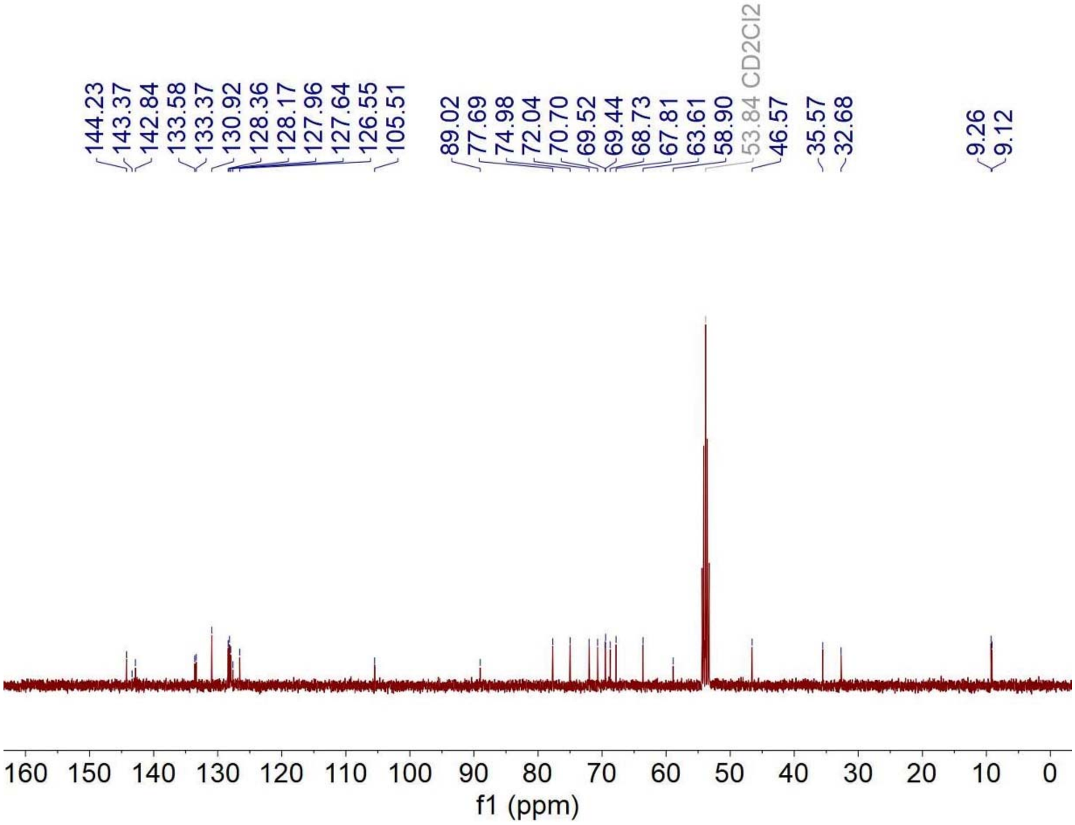

Figure S5. $^{13}C\{^{1}H\}$ NMR spectrum (101 MHz, CD_2Cl_2) of **2**.

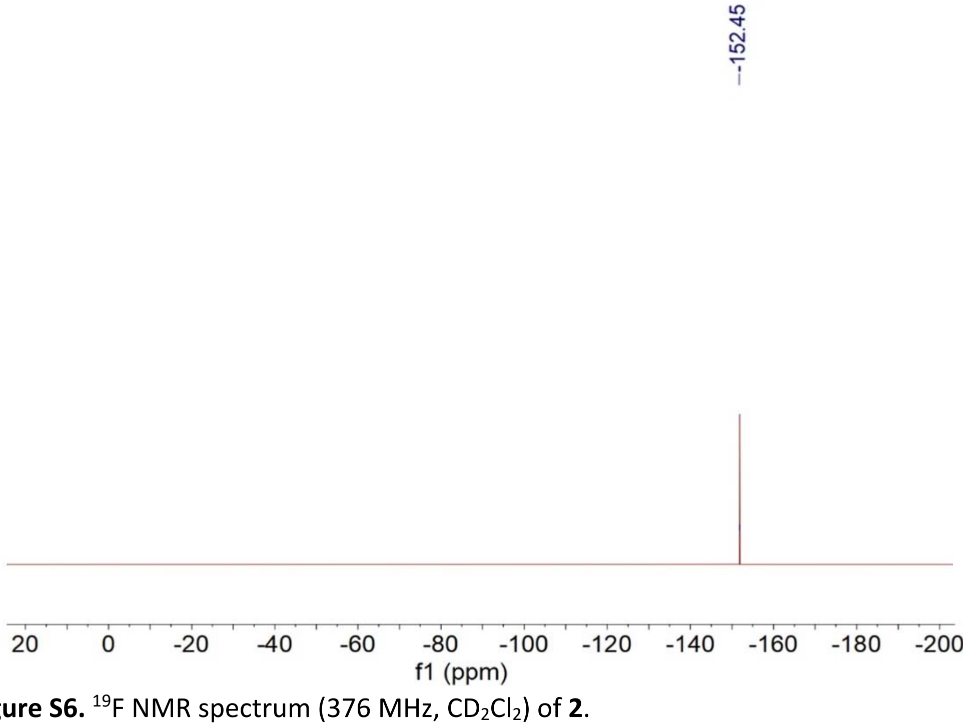

Figure S6. ^{19}F NMR spectrum (376 MHz, CD_2Cl_2) of **2**.

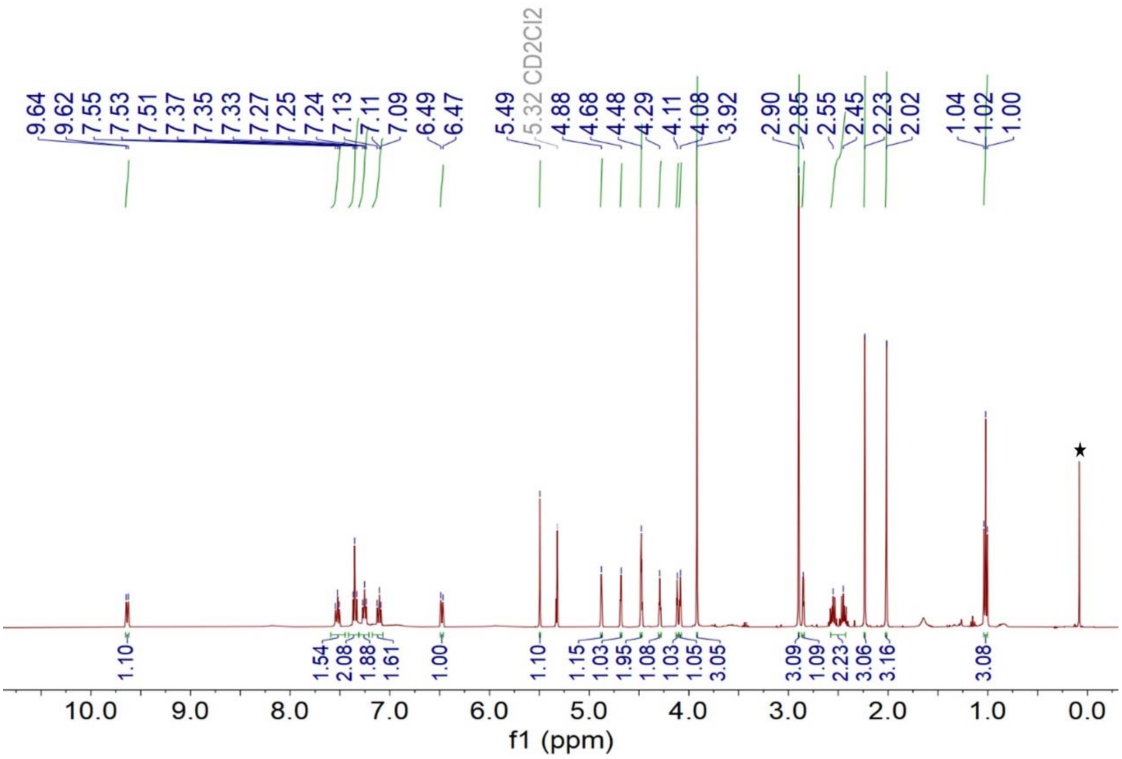

Figure S7. ^{1}H NMR spectrum (400 MHz, CD_2Cl_2) of **3**. The signal marked belongs to silicon grease (*).

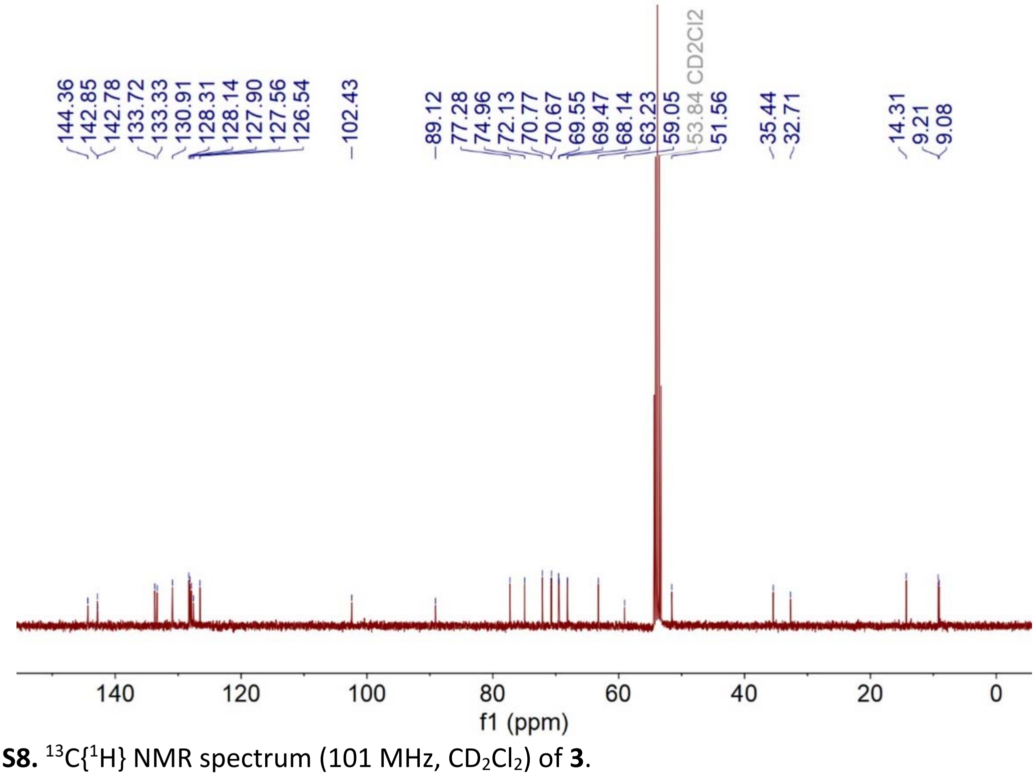

Figure S8. $^{13}C\{^{1}H\}$ NMR spectrum (101 MHz, CD_2Cl_2) of **3**.

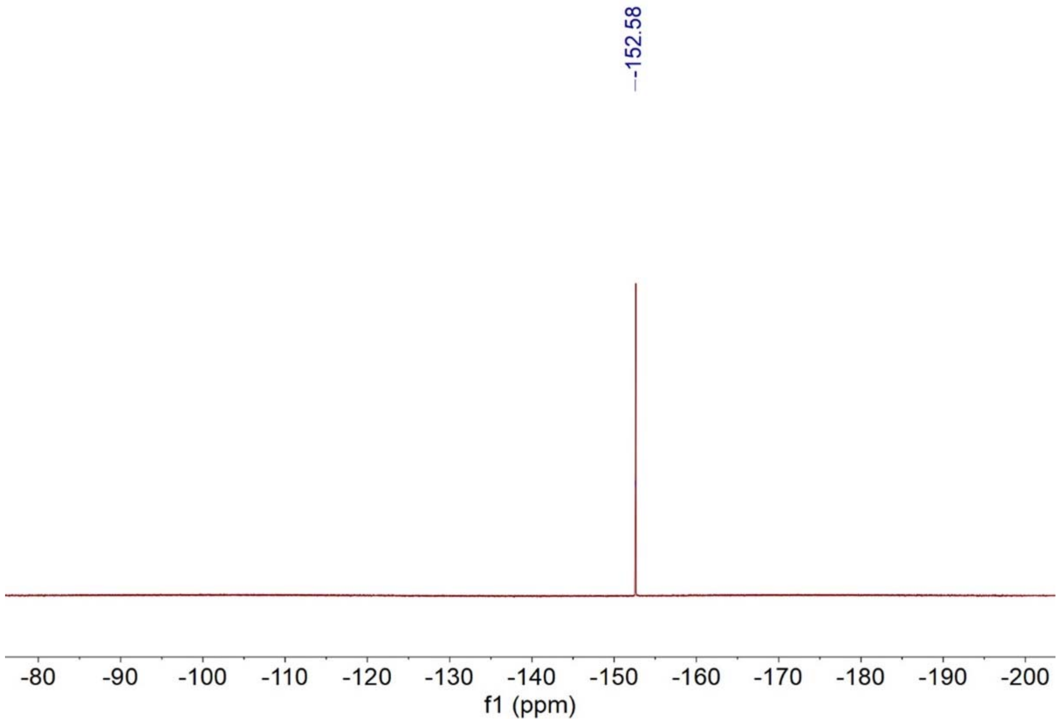

Figure S9. ^{19}F NMR spectrum (376 MHz, CD_2Cl_2) of **3**.

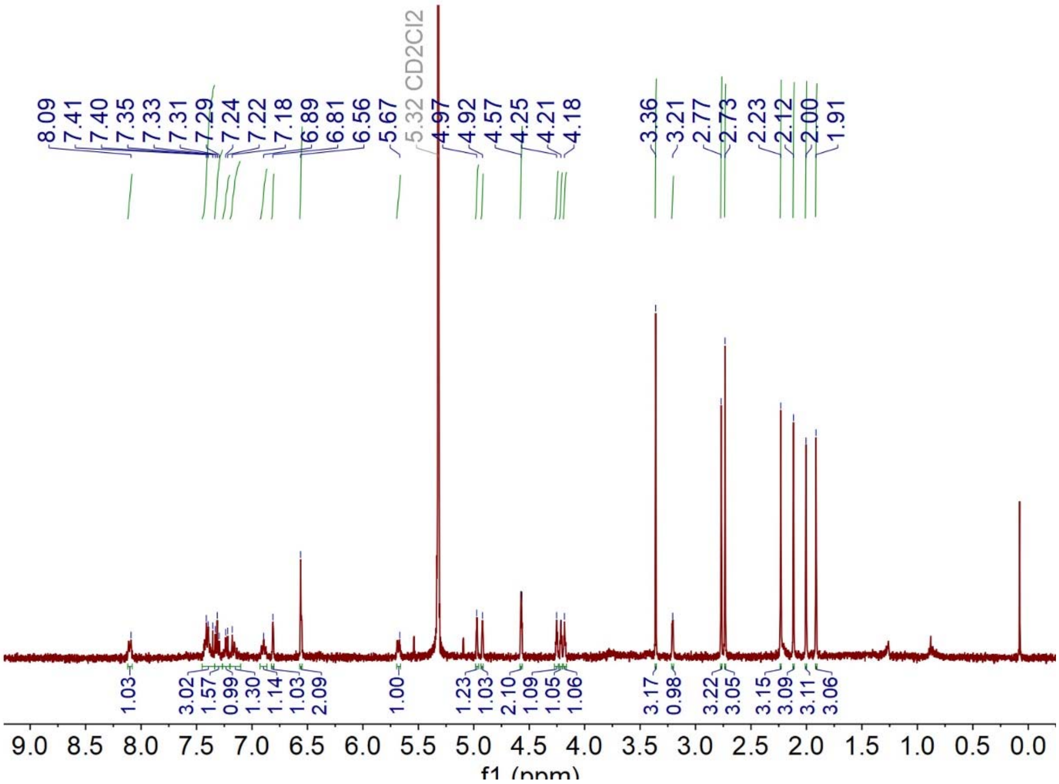

Figure S10. ^{1}H NMR spectrum (400 MHz, CD_2Cl_2) of **4**.

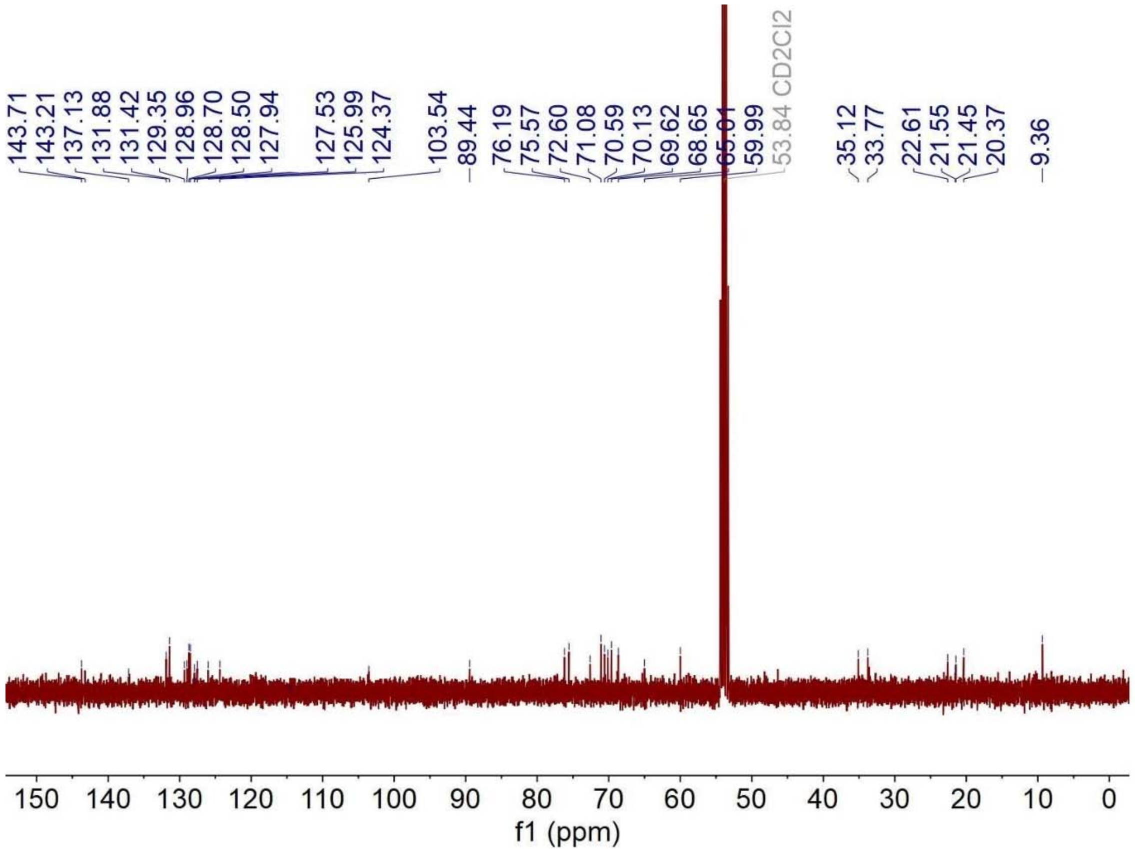

Figure S11. $^{13}C\{^{1}H\}$ NMR spectrum (101 MHz, CD_2Cl_2) of **4**.

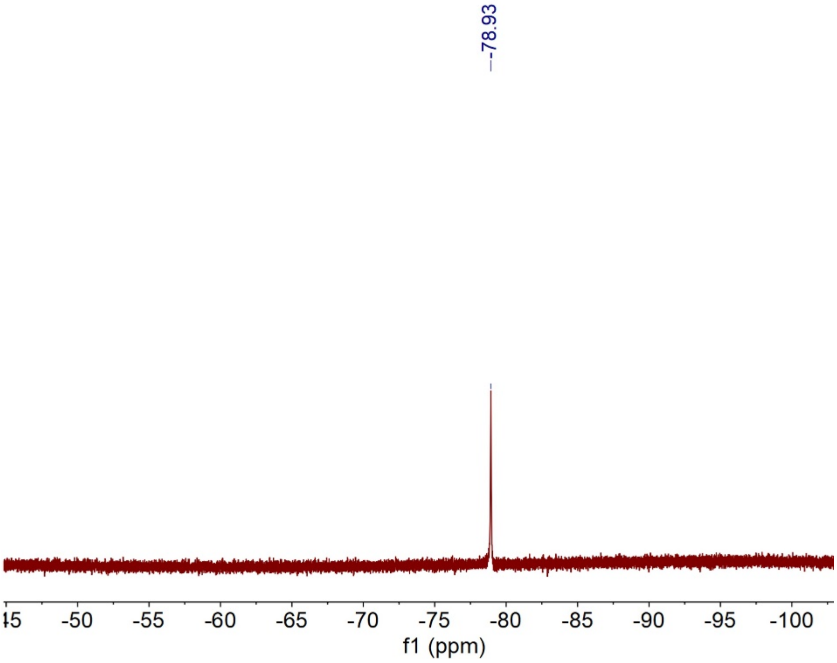

Figure S12. ^{19}F NMR spectrum (376 MHz, CD_2Cl_2) of **4**.

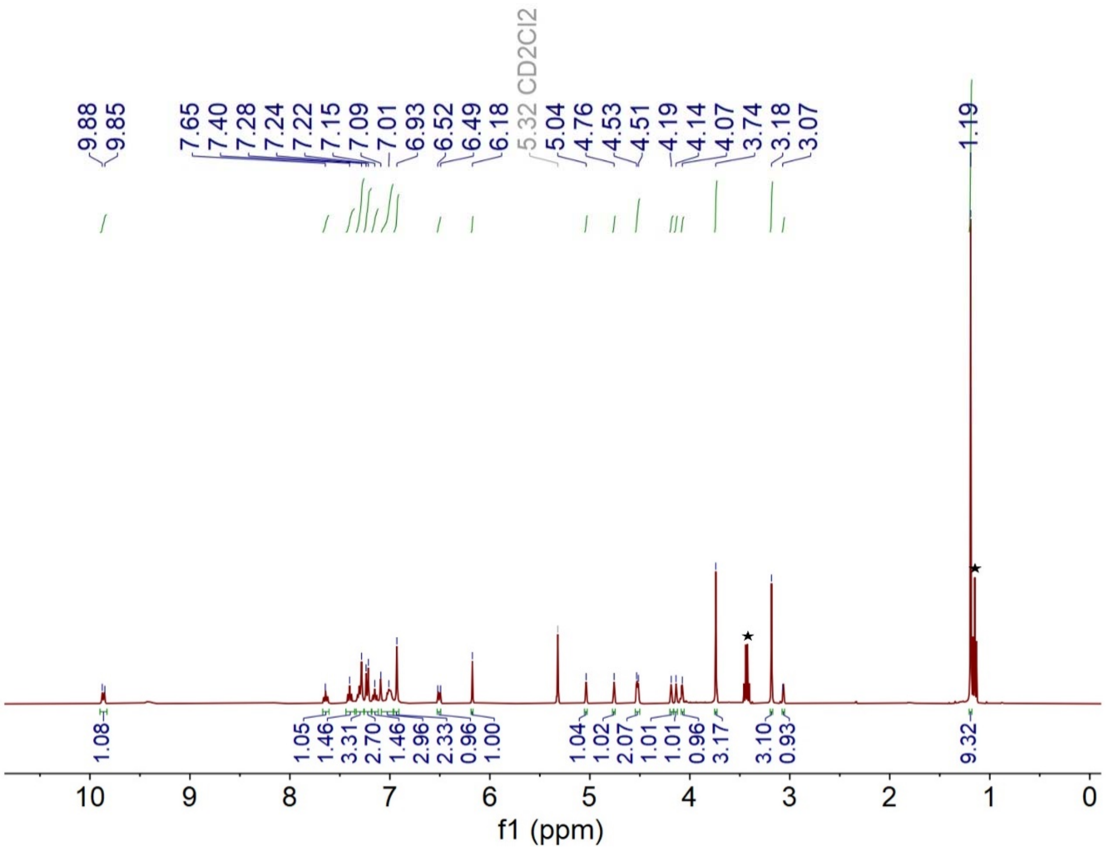

Figure S13. ^{1}H NMR spectrum (400 MHz, CD_2Cl_2) of **5**. The signals marked (*) belong to diethyl ether.

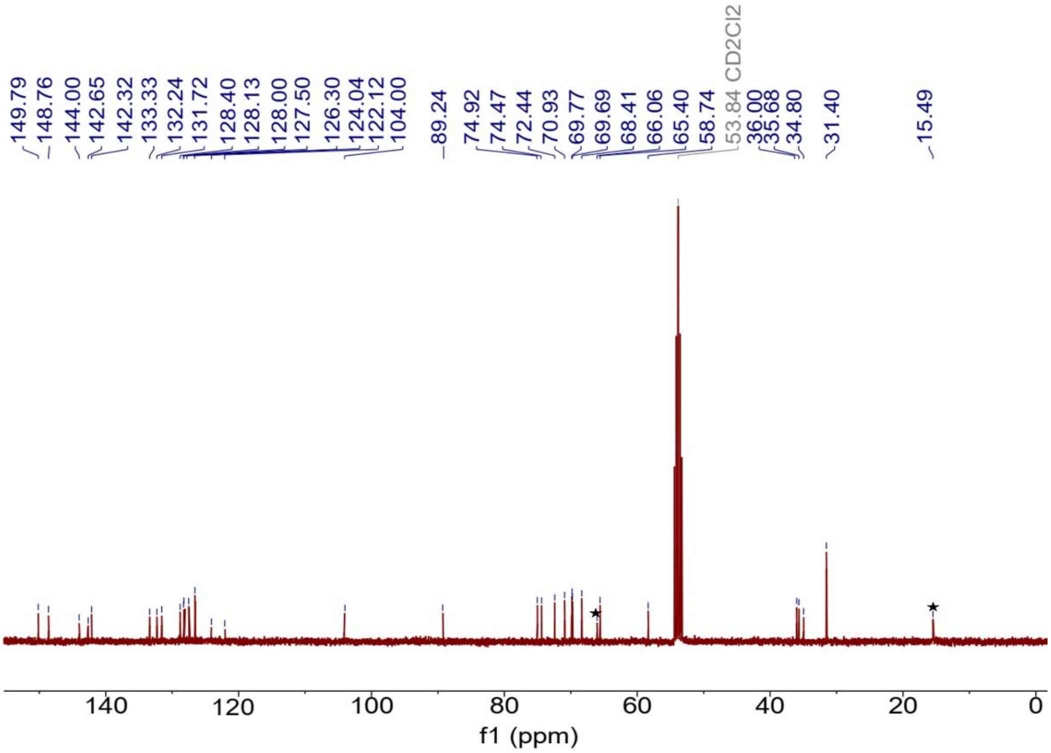

Figure S14. $^{13}C\{^{1}H\}$ NMR spectrum (101 MHz, CD_2Cl_2) of **5**. The signals marked (*) belong to diethyl ether.

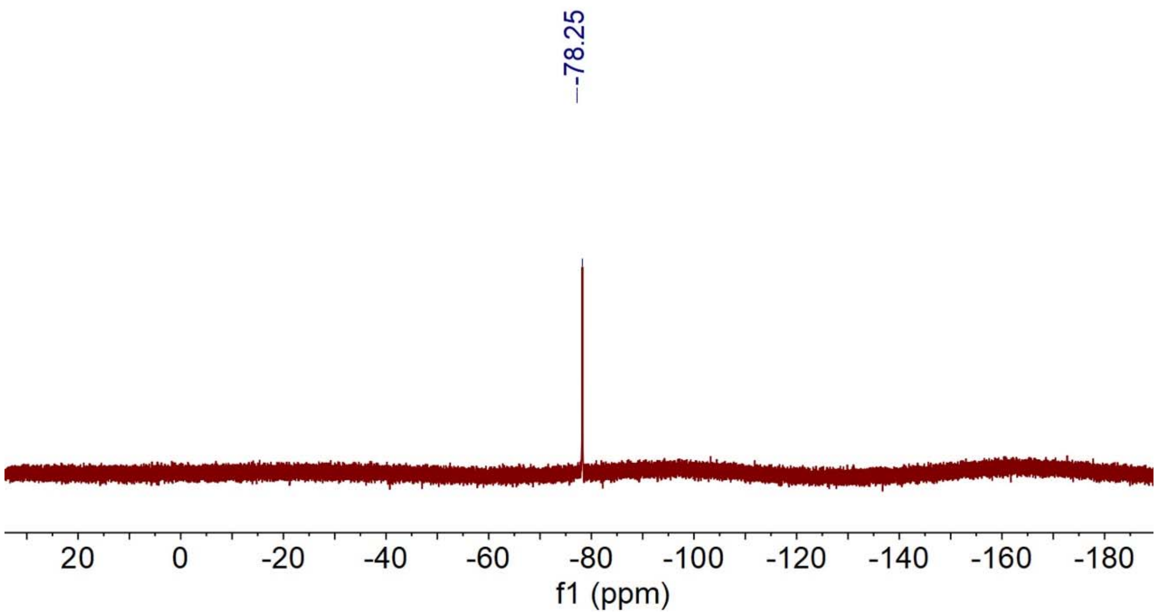

Figure S15. ^{19}F NMR spectrum (376 MHz, CD_2Cl_2) of **5**.

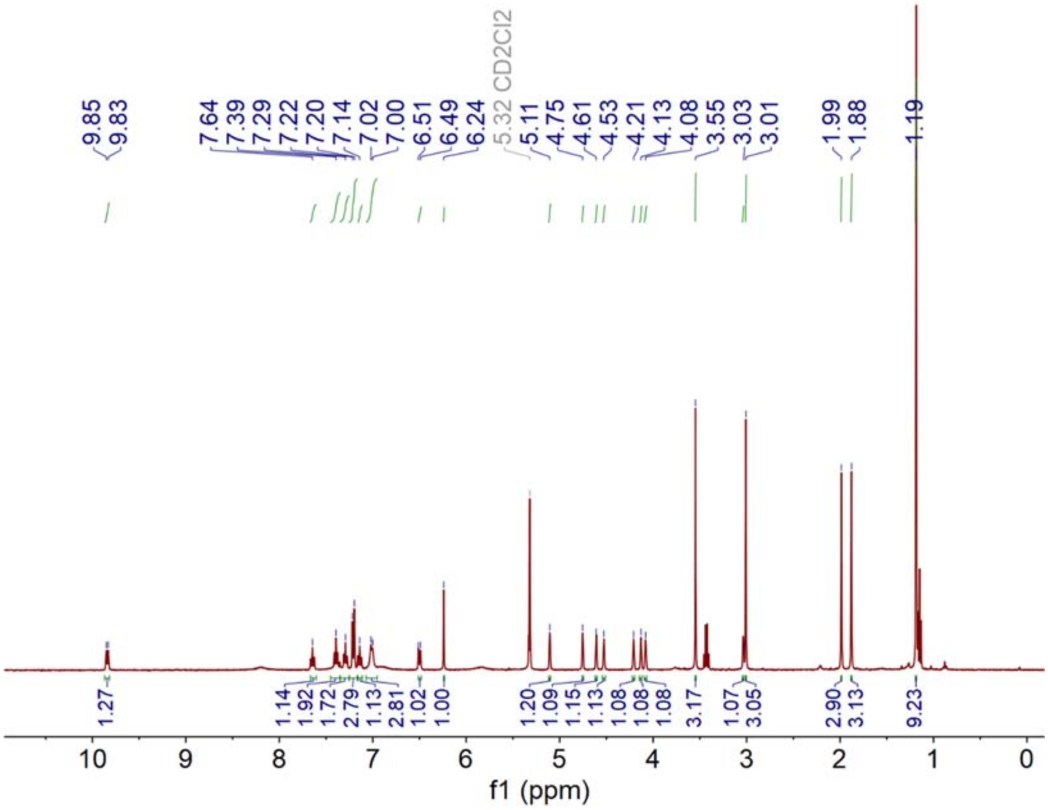

Figure S16. ^{1}H NMR spectrum (400 MHz, CD_2Cl_2) of **6**.

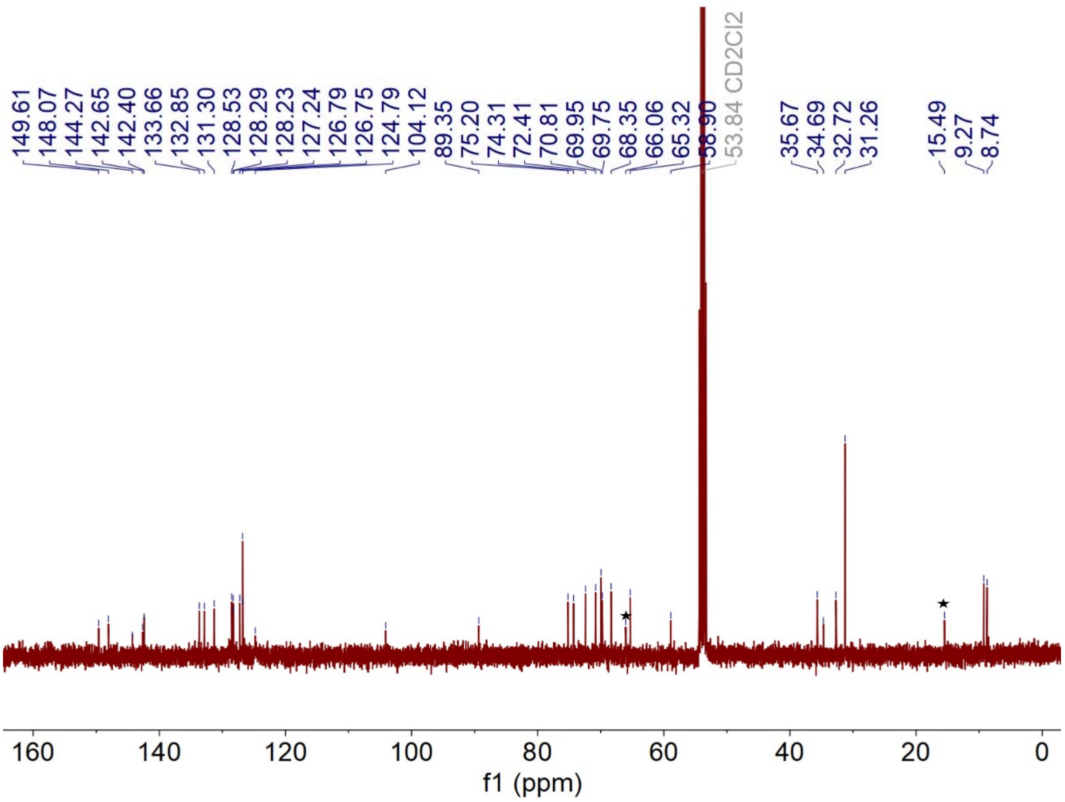

Figure S17. $^{13}C\{^1H\}$ NMR spectrum (101 MHz, CD_2Cl_2) of **6**. The signals marked (*) belong to diethyl ether.

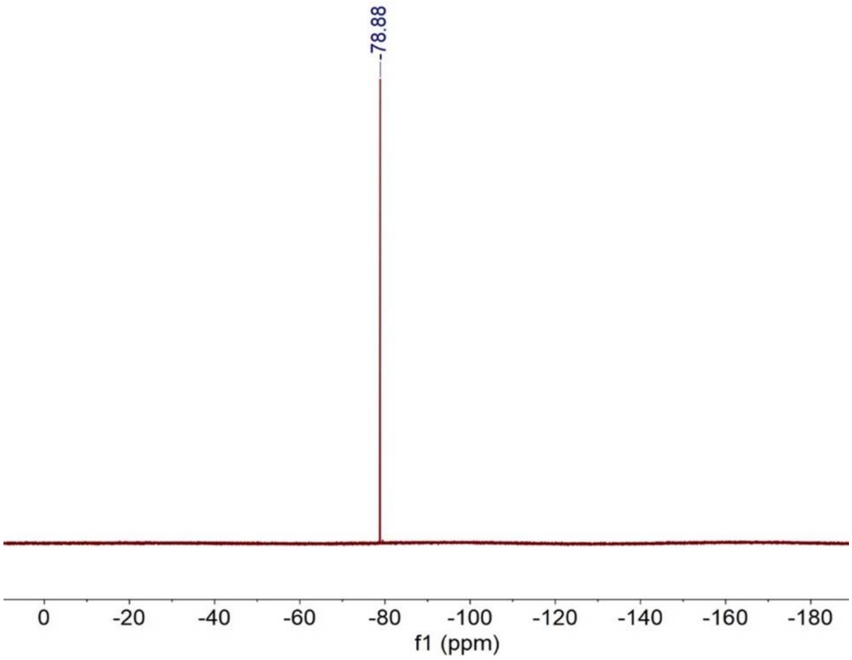

Figure S18. ^{19}F NMR spectrum (376 MHz, CD_2Cl_2) of **6**.

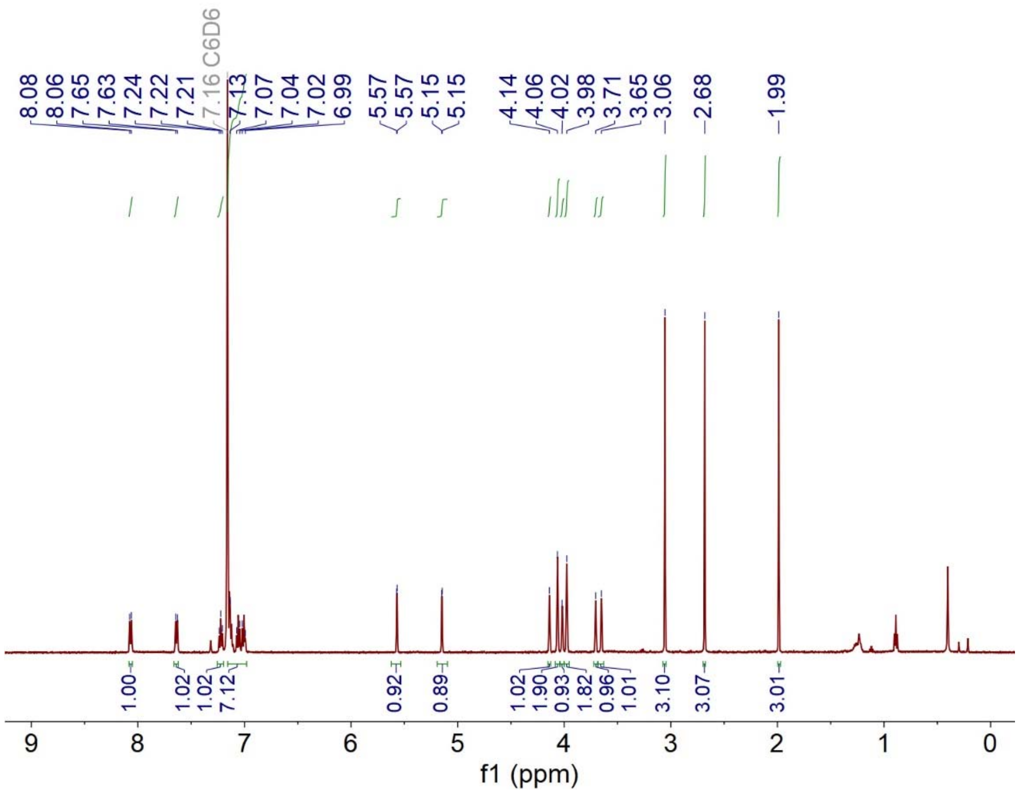

Figure S19. ^{1}H NMR spectrum (500 MHz, C_6D_6) of **7**.

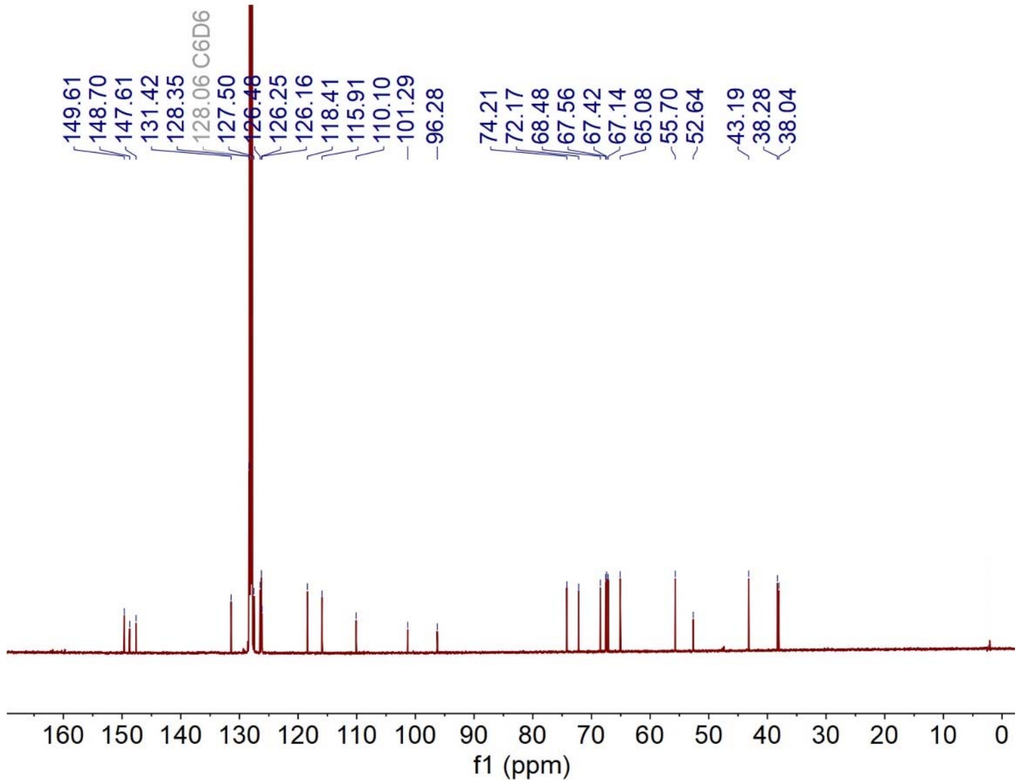

Figure S20. $^{13}C\{^{1}H\}$ NMR spectrum (126 MHz, C_6D_6) of **7**.

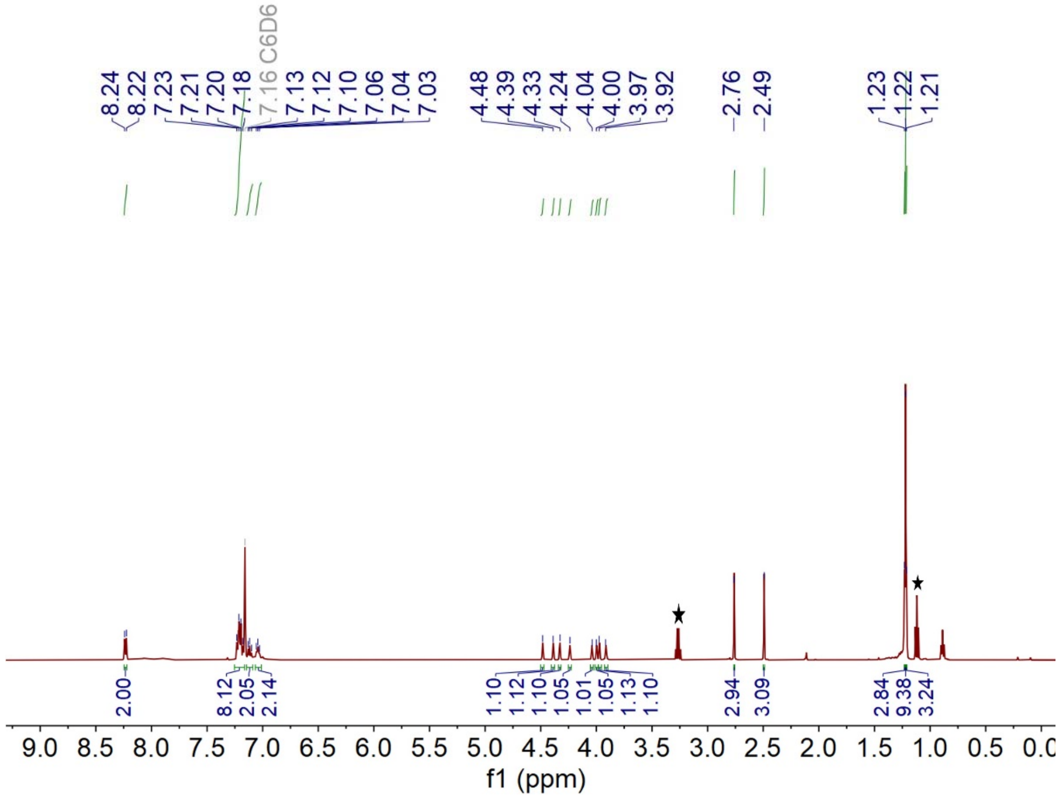

Figure S21. ^{1}H NMR spectrum (500 MHz, C_6D_6) of **8**. The signals marked (*) belong to diethyl ether.

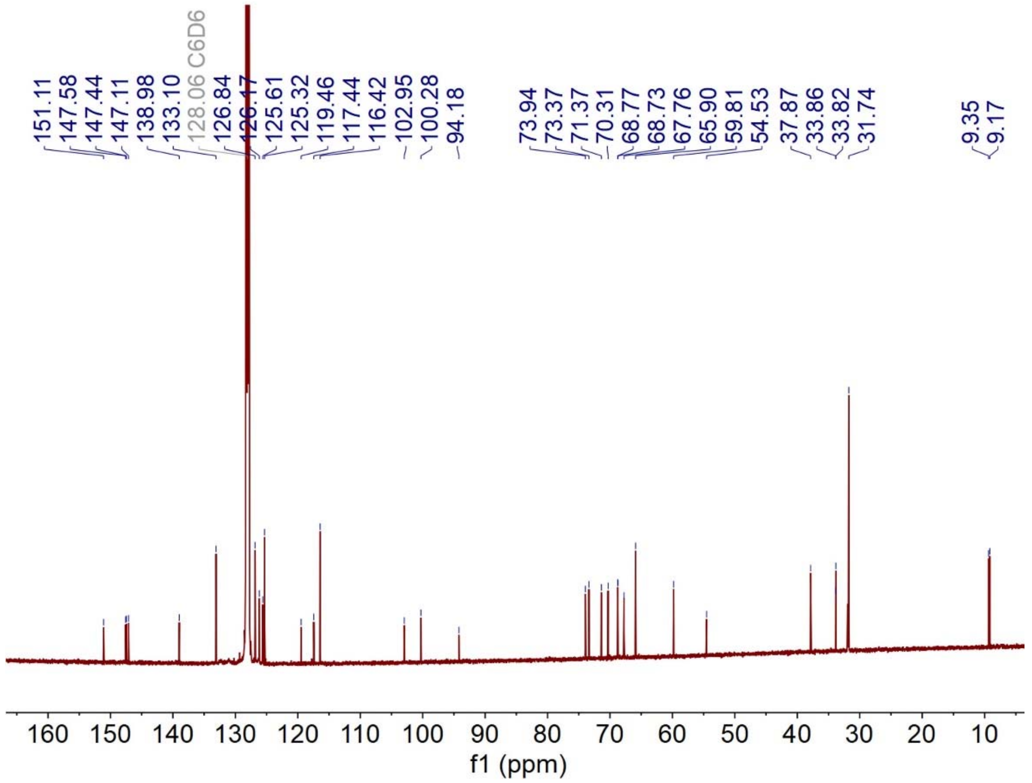

Figure S22. $^{13}C\{^{1}H\}$ NMR spectrum (126 MHz, C_6D_6) of **8**.

C Electrochemistry

Cyclic voltammograms were recorded with a *PalmSens4* potentiostat with a conventional three-electrode configuration consisting of a glassy carbon working electrode, a coiled platinum wire as counter electrode, and a coiled silver wire as a pseudo reference electrode. Ferrocene/ferrocenium was used as an internal standard. All measurements were performed at room temperature with a scan rate between 25 and 1000 mV s^{-1}. The experiments were carried out inside a glove box (*GS Glovebox Systemtechnik, GS095218*) in absolute tetrahydrofuran/acetonitrile taken from a solvent system (*MBRAUN MB-SPS-800*) containing 0.1 M nBu$_4$N[BAr^F] as the supporting electrolyte.

D Electron Paramagnetic Resonance Spectroscopy

EPR spectra were recorded at X-band frequencies using a *Magnettech MS-5000* benchtop EPR spectrometer equipped with a rectangular TE201 cavity. Spectral simulations were performed with *EasySpin* 6.0.0 integrated into *MATLAB* (R2022b), and the results were visualised using *Spyder* 5.4.3 within the *Python* 3.11 environment. Compound **8** was oxidised using ferrocenium hexafluorophosphate as oxidising agent in acetonitrile under an inert atmosphere.

E DFT Calculations

Theoretical calculations were performed using ORCA 5.0.2.[1] Geometry optimisations and frequencies were calculated starting from experimentally determined molecular structures with the PBE0 functional[2] and def2-TZVP[3] basis sets. The optimised geometries were employed for single-point and spin-density calculations with the PBE0[2] functional and def2-TZVP[3] basis sets. Implicit solvation was approximated using the SMD[4] method together with the CPCM[5] model. THF was used as the solvent of choice unless stated otherwise. Population analysis were determined from Löwdin reduced orbital populations.[6] Resolution-of-the-identity (RI) approximations[7] with matching basis-sets (def2/J)[8] were employed to reduce time of calculation. Grimme's D3 method was used for dispersion correction.[9] The visualisation software *Chemcraft* was used to plot orbital and electron/spin density figures.[10]

Table S2. Energies and compositions of selected orbitals for the native state of **7**.

**MO**	**Energy [eV]**	**Contribution [%]**				
		**C1**	**N1**	**-CH$_3$**	**Fe**	**NHC**
**HOMO-1**	-5.4386	3.3	16.5	4.2	52.9	2.1
**HOMO**	-4.2370	27.9	5.8	2.3	1.0	45.7
**LUMO**	-0.3097	0	0	0	2.2	1.1

Table S3. Energies and compositions of selected orbitals for the singly oxidised state of **7**.

MO	Energy [eV]	Contribution [%]				
		C1	N1	$-CH_3$	Fe	NHC
SOMO-α	-6.2836	30.6	20.6	4.4	2.7	7.0
SOMO-β	-6.2697	0.6	0.2	0	78.4	0
LUMO-β	-4.2370	28.1	13.4	4.4	0.3	9.0
LUMO-α	-3.9604	4.5	1.5	0.3	1.1	6.1

Table S4. Energies and compositions of selected orbitals for the doubly oxidised state of **7**.

MO	Energy [eV]	Contribution [%]				
		C1	N1	$-CH_3$	Fe	NHC
SOMO-β	-7.9322	3.3	12.2	0.1	2.1	2.8
SOMO-α	-7.3382	25.4	13.9	3.8	2.7	17.7
LUMO-β	-4.6655	16.1	4.6	0.2	34.2	7.1
LUMO-α	-2.9052	0.5	1.0	0.1	45.0	0

Table S5. Energies and compositions of selected orbitals for the native state of **8**.

MO	Energy [eV]	Contribution [%]				
		C1	N1	-*p*-C_6H_4-*t*Bu	Fe	NHC
HOMO-1	-5.6504	4.4	11.4	34.8	14.9	16.7
HOMO	-4.0214	24.8	8.5	14.5	0.8	37.6
LUMO	-0.3674	0.5	0	1.0	1.5	1.2

Table S6. Energies and compositions of selected orbitals for the singly oxidised state of **8**.

MO	Energy [eV]	Contribution [%]				
		C1	N1	-*p*-C_6H_4-*t*Bu	Fe	NHC
**SOMO-β**	-6.4503	0.9	0.2	0.3	77.2	0.7
**SOMO-α**	-5.9166	25.9	16.9	15.9	2.4	17.2
**LUMO-β**	-3.7114	3.1	0.8	20.7	1.5	11.4
**LUMO-α**	-1.1099	3.6	0.7	22.2	1.9	24.0

Table S7. Energies and compositions of selected orbitals for the doubly oxidised state of **8**.

MO	Energy [eV]	Contribution [%]				
		C1	N1	-*p*-C_6H_4-*t*Bu	Fe	NHC
**SOMO-β**	-7.2009	3.4	5.6	45.1	0.4	31.7
**SOMO-α**	-6.6158	3.6	1.8	19.9	0.2	17.9
**LUMO-β**	-4.5706	1.6	0.3	0.1	68.8	0.3
**LUMO-α**	-3.0453	3.3	5.7	46.2	0.5	33.8

Table 8. Optimised molecular structure of native **7** (left) and singly oxidised **7** (right) in Cartesian coordinates.

Atom	x	y	z	Atom	x	y	z
C	5.92274	2.42058	3.03862	C	5.90686	2.45514	3.12274
Fe	4.82227	5.41334	1.95492	Fe	5.2209	5.38697	1.73919
N	5.93358	2.71116	1.64757	N	6.12442	2.55191	1.76459
C	6.31051	3.53639	4.04709	C	6.14855	3.61553	4.10216
N	5.19835	0.15369	2.42642	N	4.34469	0.536	3.17419
N	6.00411	0.31791	4.45746	N	6.09704	0.28862	4.41175
C	5.88644	4.94707	3.58581	C	5.3883	4.8805	3.65656

C	6.62958	5.82336	2.73503	C	5.94818	6.18327	3.44322
H	7.56923	5.58314	2.26133	H	6.98009	6.46048	3.57406
C	5.90755	7.03754	2.57312	C	4.94033	7.04078	2.93496
H	6.2131	7.87938	1.96958	H	5.07843	8.07273	2.64973
C	4.71211	6.9349	3.33419	C	3.73977	6.29511	2.83595
H	3.94009	7.68539	3.41764	H	2.79185	6.65092	2.46129
C	4.70324	5.66225	3.96154	C	4.0068	4.97555	3.28524
H	3.91418	5.28114	4.58997	H	3.28424	4.17689	3.26877
C	5.01008	3.60267	1.13344	C	5.91365	3.71768	0.99396
C	5.26804	4.60904	0.1496	C	6.85641	4.76034	0.72955
H	6.20641	4.76585	-0.35911	H	7.86228	4.80914	1.11664
C	4.10897	5.42601	0.02715	C	6.19354	5.7487	-0.04138
H	4.00306	6.2759	-0.63051	H	6.62089	6.68616	-0.36383
C	3.1247	4.92767	0.92042	C	4.86498	5.30839	-0.29219
H	2.13566	5.334	1.0713	H	4.111	5.85144	-0.84185
C	3.66942	3.79859	1.59418	C	4.69061	4.04554	0.3257
H	3.18996	3.24246	2.38361	H	3.78627	3.45707	0.33811
C	5.73376	1.07838	3.32546	C	5.48266	1.14956	3.57307
C	5.10866	-1.0924	3.02101	C	4.2422	-0.68958	3.77442
H	4.7073	-1.93733	2.48784	H	3.40007	-1.33857	3.60362
C	5.59208	-0.99342	4.26256	C	5.33505	-0.84011	4.55702
H	5.71833	-1.73513	5.03246	H	5.64305	-1.6535	5.19187
C	4.48274	0.43408	1.20581	C	3.33312	1.10715	2.31168
H	3.74276	1.22179	1.35411	H	3.41221	2.1902	2.34168
H	3.96281	-0.4797	0.91824	H	2.35308	0.80439	2.67471
H	5.14523	0.73427	0.39489	H	3.46428	0.76144	1.28613
C	6.69304	0.65387	5.67946	C	7.42875	0.41559	4.96795
H	7.36484	1.49058	5.53281	H	7.87741	1.34002	4.6191
H	7.29048	-0.20935	5.97858	H	8.03047	-0.42884	4.632
H	5.99584	0.89893	6.48335	H	7.37652	0.42479	6.05467
C	7.83913	3.60935	4.27314	C	7.64315	3.96949	4.18726
C	8.43106	4.72633	4.87038	C	8.06785	4.94867	5.08835

H	7.81265	5.56579	5.16377	H	7.34057	5.44477	5.7199
C	9.79894	4.79772	5.08076	C	9.4027	5.29729	5.19793
H	10.22247	5.67962	5.54873	H	9.69893	6.05996	5.90903
C	10.62405	3.75222	4.68661	C	10.3566	4.67246	4.40425
H	11.69536	3.80862	4.84308	H	11.4026	4.94372	4.48815
C	10.05798	2.64216	4.08133	C	9.95306	3.69893	3.50715
H	10.68414	1.81876	3.7556	H	10.6809	3.19855	2.87858
C	8.68466	2.57813	3.87936	C	8.61024	3.35263	3.40297
H	8.26081	1.70846	3.39477	H	8.33002	2.59004	2.68979
C	5.55102	3.33266	5.37665	C	5.61088	3.16949	5.48533
C	6.07665	3.69219	6.61458	C	6.43625	2.95602	6.58481
H	7.08735	4.06999	6.68726	H	7.5018	3.11884	6.50679
C	5.34054	3.54559	7.78175	C	5.92506	2.51536	7.79953
H	5.78602	3.82528	8.7302	H	6.60084	2.36323	8.63353
C	4.05059	3.03714	7.74104	C	4.57147	2.26462	7.94318
H	3.47712	2.91533	8.65295	H	4.17175	1.91872	8.88934
C	3.50801	2.68531	6.51353	C	3.73459	2.45879	6.85299
H	2.50019	2.28853	6.45633	H	2.67173	2.26299	6.93767
C	4.24934	2.8357	5.35098	C	4.24943	2.90516	5.64738
H	3.81984	2.55082	4.39983	H	3.56682	3.03311	4.82115
C	7.16531	2.5608	0.9079	C	6.49599	1.38029	0.98628
H	7.87049	3.39184	1.05191	H	7.20252	1.68968	0.21443
H	6.94717	2.47997	-0.16086	H	5.63373	0.92087	0.49374
H	7.65781	1.64003	1.22135	H	6.98156	0.64131	1.62332

Table S9. Optimised molecular structure of doubly oxidised **7** in Cartesian coordinates. Left: In singlet state. Right: In triplet state.

Atom	x	y	z	Atom	x	y	z
C	5.88955	2.47157	3.10489	C	5.93728	2.45654	3.08454
Fe	5.17853	5.43441	1.75253	Fe	5.03651	5.49545	1.87214
N	6.08887	2.58119	1.74493	N	5.95294	2.65097	1.71612

C	6.16991	3.61588	4.09783	C	6.2528	3.58496	4.09739
N	4.41997	0.48512	3.16877	N	4.86991	0.25076	2.85205
N	6.16891	0.32353	4.42679	N	6.31692	0.33424	4.46155
C	5.48217	4.93187	3.67729	C	5.78163	4.98643	3.66891
C	6.14612	6.17185	3.38334	C	6.60748	6.00488	3.08662
H	7.21075	6.33438	3.38921	H	7.63848	5.8902	2.79069
C	5.17772	7.13698	3.00886	C	5.83328	7.17567	2.9102
H	5.38629	8.14726	2.68989	H	6.17515	8.08981	2.44698
C	3.911	6.52537	3.07296	C	4.52615	6.9127	3.36773
H	2.96936	6.97896	2.79879	H	3.68739	7.59171	3.32792
C	4.08434	5.17892	3.46993	C	4.48487	5.57512	3.83678
H	3.28077	4.46658	3.5472	H	3.61112	5.08142	4.2295
C	5.77383	3.71588	0.983	C	5.27322	3.68308	1.07195
C	6.68566	4.73191	0.54852	C	5.87969	4.64396	0.19722
H	7.72994	4.79408	0.81731	H	6.93201	4.7015	-0.03785
C	5.95506	5.66297	-0.22588	C	4.86512	5.51586	-0.26365
H	6.3489	6.57659	-0.64591	H	5.01417	6.37344	-0.90301
C	4.6093	5.23682	-0.2915	C	3.6396	5.1183	0.31107
H	3.80441	5.76461	-0.78178	H	2.69094	5.61962	0.18799
C	4.48123	4.03322	0.44175	C	3.87112	3.99113	1.13351
H	3.57498	3.46757	0.58868	H	3.13059	3.4948	1.73947
C	5.536	1.14197	3.5617	C	5.74269	1.08449	3.48589
C	4.34707	-0.72221	3.8048	C	4.90505	-0.9844	3.4258
H	3.52317	-1.39864	3.65137	H	4.28001	-1.78947	3.07857
C	5.441	-0.8212	4.59487	C	5.80508	-0.93152	4.43276
H	5.76784	-1.60808	5.25351	H	6.13484	-1.68658	5.12611
C	3.38278	1.00518	2.30196	C	3.88078	0.61207	1.85593
H	3.22879	2.06134	2.51262	H	3.29122	1.45068	2.21773
H	2.46347	0.46345	2.50941	H	3.22384	-0.24343	1.71945
H	3.64952	0.87091	1.2541	H	4.3431	0.85901	0.90372
C	7.47596	0.49583	5.0312	C	7.33913	0.67201	5.43738
H	7.86132	1.48556	4.8189	H	7.78216	1.63182	5.21545

H	8.15453	-0.25182	4.62061	H	8.11361	-0.09287	5.3971
H	7.38844	0.36623	6.10816	H	6.89152	0.69421	6.42956
C	7.67719	3.91734	4.19663	C	7.77909	3.73582	4.27067
C	8.12875	4.88157	5.10222	C	8.28863	4.71221	5.13024
H	7.41929	5.40699	5.73078	H	7.61306	5.34691	5.69071
C	9.47361	5.19031	5.21156	C	9.65312	4.90836	5.25993
H	9.79221	5.93879	5.9276	H	10.0206	5.66838	5.93945
C	10.40601	4.54622	4.40905	C	10.54358	4.14569	4.51577
H	11.45944	4.78589	4.49381	H	11.61139	4.30297	4.61261
C	9.97357	3.59641	3.49959	C	10.05251	3.18924	3.64342
H	10.68495	3.08341	2.86283	H	10.73124	2.58929	3.04839
C	8.62141	3.28733	3.39591	C	8.68245	2.99105	3.5228
H	8.3242	2.53452	2.67989	H	8.3321	2.22518	2.84482
C	5.56814	3.18182	5.45821	C	5.47857	3.24605	5.38874
C	6.3193	3.05964	6.62151	C	6.01721	3.33064	6.66613
H	7.37558	3.28615	6.62031	H	7.0567	3.58926	6.81008
C	5.74232	2.61717	7.80571	C	5.241	3.05285	7.78437
H	6.3586	2.53357	8.6934	H	5.68797	3.12296	8.76914
C	4.40247	2.27184	7.85131	C	3.91349	2.68131	7.64884
H	3.9558	1.91971	8.77372	H	3.31236	2.46662	8.52448
C	3.64205	2.37285	6.6945	C	3.36668	2.57706	6.37759
H	2.59478	2.09429	6.69947	H	2.33321	2.27779	6.24749
C	4.21988	2.82166	5.52032	C	4.14333	2.85448	5.26627
H	3.60092	2.855	4.63518	H	3.69332	2.76119	4.28433
C	6.55769	1.44577	0.95492	C	6.7937	1.83274	0.84413
H	7.21199	1.81915	0.16617	H	7.70788	2.3709	0.57876
H	5.73306	0.90187	0.48784	H	6.25379	1.5802	-0.06864
H	7.12926	0.76702	1.5868	H	7.0716	0.91463	1.35806

Table S10. Optimised molecular structure of native **8** (left) and singly oxidised **8** (right) in Cartesian coordinates.

Atom	x	y	z	Atom	x	y	z
C	10.87154	4.31767	8.8351	C	10.7116	4.40336	8.89572
N	10.76193	5.59057	8.1884	N	10.49632	5.62491	8.26554
Fe	11.4719	7.06419	10.69134	Fe	11.30456	7.11567	10.74079
C	10.37654	4.1177	10.2877	C	10.44469	4.15922	10.395
N	10.98498	1.96708	7.98464	N	10.74737	2.12497	7.80801
C	10.83601	5.25161	11.24361	C	11.1482	5.21184	11.27627
N	12.33436	3.41729	7.06752	N	12.34896	3.47252	7.29205
C	12.14904	5.49339	11.76178	C	12.52752	5.59991	11.22403
H	13.04023	4.9369	11.52907	H	13.28359	5.2131	10.56226
C	12.11403	6.64479	12.59294	C	12.74086	6.65979	12.14284
H	12.96158	7.08125	13.10047	H	13.67579	7.17691	12.29737
C	10.7805	7.1303	12.61236	C	11.51044	6.93694	12.78589
H	10.42439	8.00415	13.13727	H	11.33536	7.70196	13.52719
C	9.99601	6.27462	11.79346	C	10.53538	6.04939	12.26688
H	8.94807	6.40486	11.57894	H	9.49223	6.06151	12.53231
C	11.30218	6.7664	8.7526	C	10.69298	6.8815	8.90033
C	12.66348	7.07429	9.06696	C	11.91413	7.63056	8.89827
H	13.50814	6.41769	8.95204	H	12.84385	7.33235	8.43947
C	12.69494	8.3568	9.67339	C	11.68863	8.81601	9.63942
H	13.57496	8.85228	10.05626	H	12.42532	9.57749	9.84644
C	11.36914	8.86758	9.70453	C	10.33764	8.81792	10.0806
H	11.06573	9.81774	10.11781	H	9.87053	9.5755	10.69151
C	10.51031	7.89494	9.1348	C	9.70932	7.63953	9.60622
H	9.43768	7.96266	9.03158	H	8.69376	7.32769	9.79512
C	11.04505	2.82582	10.82743	C	11.04566	2.77469	10.76156
C	10.35789	1.80143	11.4697	C	10.28903	1.72177	11.26474
H	9.28461	1.852	11.58091	H	9.224	1.8345	11.4074
C	11.01143	0.67279	11.95383	C	10.86467	0.49366	11.57128

H	10.43423	-0.10326	12.44443	H	10.23868	-0.29896	11.96541
C	12.38011	0.53586	11.80521	C	12.21518	0.27851	11.36396
H	12.89252	-0.34231	12.18168	H	12.66407	-0.68006	11.59694
C	13.08317	1.53843	11.14949	C	12.98392	1.31061	10.84269
H	14.15367	1.44652	11.00157	H	14.04201	1.1651	10.65766
C	12.42534	2.65911	10.67271	C	12.40678	2.53587	10.55508
H	12.99024	3.40545	10.13011	H	13.04002	3.30491	10.13835
C	8.84433	4.08629	10.44867	C	8.93941	4.21087	10.70613
C	8.25958	3.90312	11.70631	C	8.49072	3.95103	12.0038
H	8.89104	3.78616	12.57867	H	9.20898	3.72671	12.78252
C	6.88552	3.88735	11.87094	C	7.14323	3.97212	12.31899
H	6.46776	3.72941	12.85919	H	6.82814	3.75859	13.33386
C	6.04555	4.08641	10.78165	C	6.20151	4.26695	11.34097
H	4.96898	4.07671	10.90915	H	5.14545	4.28372	11.58421
C	6.60646	4.31227	9.53582	C	6.62884	4.53951	10.05322
H	5.97357	4.49331	8.67402	H	5.91063	4.77859	9.27719
C	7.98929	4.31349	9.37974	C	7.98409	4.50987	9.74219
H	8.40694	4.49654	8.39979	H	8.28288	4.73311	8.72818
C	11.36312	3.29088	8.03868	C	11.23085	3.36154	8.03955
C	11.78536	1.27399	7.07113	C	11.5742	1.44557	6.93932
C	12.61093	2.17084	6.49925	C	12.57809	2.30221	6.60117
C	9.70103	1.45119	8.38595	C	9.46884	1.60398	8.23555
H	9.11032	2.25765	8.80777	H	8.94686	2.35467	8.82069
H	9.79819	0.66386	9.13346	H	9.61029	0.71263	8.84355
H	9.17208	1.05473	7.51499	H	8.87372	1.35488	7.35574
C	11.64049	-0.18448	6.8603	C	11.30406	0.0522	6.52608
H	11.78058	-0.73344	7.79659	H	11.24073	-0.6063	7.39651
H	12.38548	-0.53732	6.14793	H	12.10428	-0.30829	5.88239
H	10.6531	-0.45058	6.47064	H	10.36152	-0.0271	5.97806
C	13.65534	1.99992	5.46526	C	13.74086	2.11193	5.70946
H	13.46919	2.64768	4.60342	H	13.76849	2.86852	4.92151
H	13.66569	0.96814	5.11571	H	13.68638	1.13242	5.23738

H	14.6554	2.23435	5.84327	H	14.68363	2.16533	6.2607
C	13.25048	4.52168	7.01868	C	13.22304	4.62332	7.26837
H	12.74939	5.44674	6.73789	H	12.83545	5.38355	6.58916
H	14.02441	4.31079	6.28536	H	14.2096	4.30813	6.94031
H	13.71627	4.65911	7.99835	H	13.29952	5.03854	8.27066
C	10.22185	5.72626	6.90892	C	10.11491	5.69717	6.9018
C	9.53475	4.69775	6.25087	C	9.38829	4.68675	6.27468
H	9.40265	3.73616	6.7241	H	9.05417	3.81851	6.82359
C	9.02034	4.87444	4.9732	C	9.04635	4.78547	4.93289
H	8.49667	4.03823	4.52593	H	8.48241	3.97175	4.4954
C	9.17053	6.06067	4.26507	C	9.38396	5.89568	4.16636
C	9.88367	7.07191	4.9113	C	10.08927	6.91036	4.81465
H	10.05471	8.01924	4.41057	H	10.37221	7.80528	4.27244
C	10.40032	6.9182	6.18238	C	10.45108	6.82244	6.14409
H	10.95598	7.73668	6.61928	H	11.00187	7.63523	6.59586
C	8.60612	6.2862	2.87044	C	8.97342	6.06596	2.71122
C	7.5534	7.39968	2.92394	C	7.96281	7.2165	2.62656
H	6.73065	7.12153	3.58738	H	7.07343	6.99198	3.22006
H	7.14368	7.58167	1.92612	H	7.65442	7.37091	1.58888
H	7.98024	8.33619	3.28991	H	8.3921	8.15096	2.99432
C	9.72858	6.71119	1.91839	C	10.19603	6.40981	1.85549
H	10.21294	7.62998	2.25485	H	10.67134	7.33732	2.17987
H	9.32739	6.89398	0.91716	H	9.89127	6.54041	0.81399
H	10.49363	5.93346	1.84386	H	10.9413	5.61123	1.89906
C	7.94637	5.03057	2.31076	C	8.32766	4.80719	2.14363
H	8.65633	4.20268	2.23541	H	9.00712	3.95159	2.18595
H	7.56163	5.2356	1.30841	H	8.0657	4.97464	1.09645
H	7.10527	4.70535	2.92843	H	7.41101	4.54593	2.67767

Table S11. Optimised molecular structure of doubly oxidised **8** in Cartesian coordinates. Left: In singlet state. Right: In triplet state.

Atom	x	y	z	Atom	x	y	z
C	10.57779	4.54281	8.89661	C	10.76913	4.37821	8.89713
N	10.13784	5.62954	8.3304	N	10.6092	5.60259	8.25463
Fe	10.54692	7.3614	10.68931	Fe	11.27106	7.13606	10.77527
C	10.7759	4.30699	10.41206	C	10.43411	4.13375	10.38559
N	9.98612	2.59402	7.37685	N	10.68181	2.07117	7.86341
C	11.17062	5.55965	11.17944	C	10.93211	5.24609	11.33263
N	12.087	3.05661	7.56484	N	12.27931	3.37241	7.22292
C	12.29345	6.41537	10.93445	C	12.28001	5.60351	11.67264
H	13.0255	6.30095	10.14922	H	13.1825	5.17768	11.268
C	12.23804	7.49405	11.85441	C	12.25136	6.66737	12.60752
H	12.9275	8.32377	11.8913	H	13.11773	7.17725	13.00314
C	11.10197	7.31034	12.68234	C	10.90589	6.99081	12.86554
H	10.77097	7.97526	13.46534	H	10.55224	7.79385	13.49551
C	10.45149	6.1174	12.28403	C	10.08951	6.13084	12.09245
H	9.53634	5.73033	12.70167	H	9.01397	6.17173	12.04906
C	9.84146	6.88009	8.96485	C	10.94359	6.83968	8.84432
C	10.68677	8.01715	8.78488	C	12.25585	7.39528	9.01601
H	11.59692	8.04137	8.2055	H	13.1909	6.91561	8.77562
C	10.14257	9.06065	9.56941	C	12.10024	8.68453	9.58172
H	10.57615	10.04127	9.6913	H	12.9042	9.34212	9.87806
C	8.95601	8.58976	10.18813	C	10.72061	8.94026	9.74549
H	8.32148	9.15171	10.85623	H	10.29214	9.81996	10.20281
C	8.74214	7.24107	9.8105	C	9.99824	7.81462	9.29149
H	7.92714	6.61123	10.1226	H	8.92676	7.6796	9.30715
C	11.80851	3.17489	10.61892	C	11.19019	2.83982	10.78489
C	11.47681	1.86705	10.2596	C	10.58383	1.73042	11.36161
H	10.49477	1.64268	9.85988	H	9.52103	1.7208	11.55253
C	12.37418	0.82944	10.43217	C	11.31805	0.59157	11.67256

H	12.09309	-0.17443	10.13653	H	10.81251	-0.25525	12.12195
C	13.61778	1.07176	11.00411	C	12.67439	0.53256	11.40367
H	14.31985	0.25937	11.15044	H	13.24479	-0.35711	11.64306
C	13.94033	2.35631	11.40464	C	13.29178	1.62535	10.8103
H	14.89631	2.55924	11.8728	H	14.34919	1.59718	10.57433
C	13.04355	3.40118	11.21124	C	12.5582	2.75935	10.51116
H	13.32298	4.39135	11.54018	H	13.06845	3.58058	10.02521
C	9.42475	3.80062	10.98202	C	8.91379	4.07241	10.62719
C	9.45714	3.10604	12.19187	C	8.42413	3.73167	11.89022
H	10.40482	2.90172	12.67393	H	9.11343	3.48216	12.6873
C	8.28522	2.68977	12.79656	C	7.06716	3.74932	12.16092
H	8.33394	2.16433	13.74307	H	6.7175	3.4767	13.14968
C	7.05604	2.94143	12.19813	C	6.16287	4.13302	11.17824
H	6.13992	2.60507	12.66876	H	5.10053	4.1539	11.39067
C	7.01541	3.62636	10.99765	C	6.63567	4.50239	9.93124
H	6.06831	3.83436	10.51414	H	5.94771	4.82182	9.15709
C	8.19434	4.05775	10.4003	C	7.99892	4.47365	9.6614
H	8.11423	4.59878	9.47056	H	8.33382	4.77634	8.68033
C	10.86358	3.42468	7.95473	C	11.20641	3.3	8.04096
C	10.6695	1.66253	6.62753	C	11.43326	1.36172	6.95402
C	11.99864	1.96088	6.74235	C	12.43423	2.18853	6.53658
C	8.5416	2.59924	7.5204	C	9.4385	1.55391	8.39324
H	8.17503	3.62035	7.53642	H	8.97781	2.2821	9.04949
H	8.25281	2.07777	8.43215	H	9.62915	0.63857	8.95003
H	8.11282	2.09826	6.65759	H	8.7592	1.34367	7.56588
C	9.99516	0.58064	5.88111	C	11.10422	-0.03026	6.58149
H	9.3839	-0.03624	6.5433	H	11.10092	-0.67639	7.46313
H	10.74167	-0.05745	5.41212	H	11.83985	-0.41499	5.87762
H	9.35108	0.98122	5.09438	H	10.11742	-0.0959	6.11595
C	13.19266	1.32041	6.15602	C	13.51532	1.95537	5.5575
H	13.718	2.0047	5.48487	H	13.49488	2.7008	4.75874
H	12.90063	0.44117	5.58573	H	13.39293	0.97174	5.10807

H	13.89053	1.01065	6.93748	H	14.50169	1.98986	6.02727
C	13.34633	3.69054	7.90163	C	13.20541	4.48067	7.15468
H	13.88573	3.90727	6.98026	H	12.78551	5.30244	6.57397
H	13.93441	3.02194	8.52782	H	14.1196	4.1403	6.67745
H	13.16898	4.6211	8.43053	H	13.4432	4.81736	8.16168
C	9.97703	5.71459	6.88902	C	10.18024	5.70312	6.90118
C	8.70334	5.89485	6.38323	C	9.39912	4.72479	6.29269
H	7.85041	5.95782	7.04931	H	9.05818	3.85637	6.83706
C	8.53474	5.98348	5.01025	C	9.02307	4.85154	4.96312
H	7.5307	6.10386	4.62703	H	8.41917	4.06276	4.53424
C	9.61922	5.92561	4.13794	C	9.37972	5.95679	4.1971
C	10.89457	5.79596	4.69461	C	10.14456	6.9369	4.82953
H	11.76745	5.78349	4.05372	H	10.44722	7.82364	4.28531
C	11.08587	5.69325	6.05853	C	10.54513	6.81955	6.14609
H	12.08536	5.63572	6.46879	H	11.15528	7.60287	6.57639
C	9.46351	6.01865	2.62823	C	8.93776	6.14991	2.75441
C	10.21963	7.2535	2.12538	C	7.95876	7.32974	2.70513
H	9.81598	8.16534	2.57202	H	7.0819	7.13107	3.32583
H	10.1185	7.32957	1.04005	H	7.62281	7.49422	1.67786
H	11.28415	7.19831	2.36149	H	8.4281	8.25029	3.05887
C	10.05819	4.75979	1.98731	C	10.14834	6.46872	1.87213
H	11.12042	4.6521	2.21538	H	10.6534	7.38184	2.19133
H	9.95162	4.81556	0.90137	H	9.82019	6.61538	0.84013
H	9.53947	3.86323	2.33619	H	10.87311	5.65073	1.89057
C	8.00318	6.13523	2.20603	C	8.24502	4.91293	2.19394
H	7.41749	5.27125	2.53018	H	8.90228	4.03935	2.21183
H	7.94841	6.18484	1.11675	H	7.96256	5.09674	1.15498
H	7.53681	7.03948	2.60453	H	7.33358	4.6706	2.74576

Table S12. Calculated final point energies for the singlet and triplet state of doubly oxidised **7** and **8** and the corresponding singlet–triplet energy gaps.

	E(Singlet) [E_H]	E(Triplet) [E_H]	ΔE_{ST} [kJ/mol]
**7**$^{2+}$	-2586.973517377514	-2587.014157634291	**106.7**
**8**$^{2+}$	-3014.300941860941	-3014.314776377522	**36.3**

F References

[1] a) F. Neese, *Wiley Interdiscip. Rev.: Comput. Mol. Sci.* **2012**, *2*, 73 – 78; b) F. Neese, *Wiley Interdiscip. Rev.: Comput. Mol. Sci.* **2022**, *12*, e1606.

[2] C. Adamo, V. Barone, *J. Chem. Phys.* **1999**, *110*, 6158 – 6170.

[3] F. Weigend, R. Ahlrichs, *Phys. Chem. Chem. Phys.* **2005**, *7*, 3297 – 3305.

[4] A. V. Marenich, C. J. Cramer, D. G. Truhlar, *J. Phys. Chem. B* **2009**, *113*, 6378 – 6396.

[5] V. Barone, M. Cossi, *J. Phys. Chem. A* **1998**, *102*, 1995 – 2001.

[6] P.-O. Löwdin, *J. Appl. Phys.* **1962**, *33*, 251 – 280.

[7] a) T. Petrenko, S. Kossmann, F. Neese, *J. Chem. Phys.* **2011**, *134*, 054116; b) F. Neese, G. Olbrich, *Chem. Phys. Lett.* **2002**, *362*, 170 – 178; c) R. Izsák, F. Neese, *J. Chem. Phys.* **2011**, *135*, 144105; d) J. L. Whitten, *J. Chem. Phys.* **1973**, *58*, 4496 – 4501; e) O. Vahtras, J. Almlöf, M. W. Feyereisen, *Chem. Phys. Lett.* **1993**, *213*, 514 – 518; f) F. Neese, F. Wennmohs, A. Hansen, U. Becker, *Chem. Phys.* **2009**, *356*, 98 – 109.

[8] a) K. Eichkorn, O. Treutler, H. Öhm, M. Häser, R. Ahlrichs, *Chem. Phys. Lett.* **1995**, *242*, 652 – 660; b) K. Eichkorn, F. Weigend, O. Treutler, R. Ahlrichs, *Theor. Chem. Acc.* **1997**, *97*, 119 – 124; c) F. Weigend, *Phys. Chem. Chem. Phys.* **2006**, *8*, 1057 – 1065.

[9] S. Grimme, J. Antony, S. Ehrlich, H. Krieg, *J. Chem. Phys.* **2010**, *132*, 154104.

[10] Chemcraft – graphical software for visualisation of quantum chemistry computations. https://www.chemcraftprog.com
